# A Cell-Based Platform for the Investigation of Immunoproteasome Subunit β5i Expression and Biology of β5i-Containing Proteasomes

**DOI:** 10.3390/cells10113049

**Published:** 2021-11-05

**Authors:** Alexander Burov, Sergei Funikov, Elmira Vagapova, Alexandra Dalina, Alexander Rezvykh, Elena Shyrokova, Timofey Lebedev, Ekaterina Grigorieva, Vladimir Popenko, Olga Leonova, Daria Spasskaya, Pavel Spirin, Vladimir Prassolov, Vadim Karpov, Alexey Morozov

**Affiliations:** 1 Laboratory of Regulation of Intracellular Proteolysis, Engelhardt Institute of Molecular Biology, Russian Academy of Sciences, 119991 Moscow, Russia; alexanderburov1998@gmail.com (A.B.); grigorieva.ev@phystech.edu (E.G.); drspssk@gmail.com (D.S.); karpovvl2008@gmail.com (V.K.); 2 Laboratory of Molecular Mechanisms of Biological Adaptation, Engelhardt Institute of Molecular Biology, Russian Academy of Sciences, 119991 Moscow, Russia; sergeifunikov@mail.ru (S.F.); aprezvykh@yandex.ru (A.R.); 3 Department of Cancer Cell Biology, Engelhardt Institute of Molecular Biology, Russian Academy of Sciences, 119991 Moscow, Russia; vr.elmira@gmail.com (E.V.); elena.j.shirokova@phystech.edu (E.S.); lebedevtd@gmail.com (T.L.); popenko@eimb.ru (V.P.); leonova-kozma@mail.ru (O.L.); discipline82@mail.ru (P.S.); prassolov45@mail.ru (V.P.); 4 Center for Precision Genome Editing and Genetic Technologies for Biomedicine, Engelhardt Institute of Molecular Biology, Russian Academy of Sciences, 119991 Moscow, Russia; alexandra.dalina@gmail.com; 5 Laboratory of Cell Proliferation, Engelhardt Institute of Molecular Biology, Russian Academy of Sciences, 119991 Moscow, Russia; 6 Moscow Institute of Physics and Technology, National Research University, Dolgoprudny, 141701 Moscow, Russia

**Keywords:** proteasome, immunoproteasome, intermediate proteasome, non-constitutive proteasome, reporter cell line

## Abstract

The degradation of most intracellular proteins is a dynamic and tightly regulated process performed by proteasomes. To date, different forms of proteasomes have been identified. Currently the role of non-constitutive proteasomes (immunoproteasomes (iPs) and intermediate proteasomes (intPs)) has attracted special attention. Here, using a CRISPR-Cas9 nickase technology, four cell lines: histiocytic lymphoma, colorectal adenocarcinoma, cervix adenocarcinoma, and hepatocarcinoma were modified to express proteasomes with mCherry-tagged β5i subunit, which is a catalytic subunit of iPs and intPs. Importantly, the expression of the chimeric gene in modified cells is under the control of endogenous regulatory mechanisms and is increased following IFN-γ and/or TNF-α stimulation. Fluorescent proteasomes retain catalytic activity and are distributed within the nucleus and cytoplasm. RNAseq reveals marginal differences in gene expression profiles between the modified and wild-type cell lines. Predominant metabolic pathways and patterns of expressed receptors were identified for each cell line. Using established cell lines, we demonstrated that anti-cancer drugs Ruxolitinib, Vincristine and Gefitinib stimulated the expression of β5i-containing proteasomes, which might affect disease prognosis. Taken together, obtained cell lines can be used as a platform for real-time studies of immunoproteasome gene expression, localization of iPs and intPs, interaction of non-constitutive proteasomes with other proteins, proteasome trafficking and many other aspects of proteasome biology in living cells. Moreover, the established platform might be especially useful for fast and large-scale experiments intended to evaluate the effects of different conditions including treatment with various drugs and compounds on the proteasome pool.

## 1. Introduction

Most proteins in cells are degraded by the ubiquitin-proteasome system (UPS). A core element of UPS is the 20S proteasome complex comprising four heptameric rings of either alpha or beta subunits arranged according to the αββα symmetry. Three of seven beta subunits (β1, β2, and β5) perform protein hydrolysis and exert caspase-like, trypsin-like, and chymotrypsin-like catalytic activities, respectively [1]. Upon inflammation and stress, constitutive catalytic subunits are replaced by the so-called immune catalytic subunits: β1i (encoded by PSMB9 gene), β2i (encoded by PSMB10), and β5i (encoded by PSMB8) [2]. Due to the structural peculiarities of the immune subunits, immunoproteasomes (iPs) demonstrate lower caspase-like activity, but higher chymotrypsin-like activity than the constitutive proteasomes, which is essential for the production of MHCI-compatible peptides [3]. Therefore, iPs play an important role in inflammation and immune response [2,4]. Along these lines, immune cells demonstrate high basal levels of the immunoproteasome subunit expression. However, iPs are assembled in many different somatic cells in response to the stimulation by cytokines and molecules including IFN-γ, TNF-α, type I interferons, IL-1β, lipopolysaccharides, and nitric oxide [5,6,7,8,9]. The accumulating evidence indicates the broader role of iPs in cellular metabolism and maintenance of homeostasis [10]. Thus, immunoproteasomes have been shown to regulate different signaling pathways and the expression of more than 8000 genes in dendritic cells [11]. IPs are involved in the degradation of oxidized and damaged proteins being upregulated as a part of the cellular response to stress [12]. Recent studies have demonstrated that immunoproteasomes participate in the maintenance of the pluripotent state of stem cells [13], T cell activation, transmission of visual signal, muscle differentiation, cytokine synthesis and, possibly, memory formation [14,15,16,17,18,19]. The level of IP subunit expression in tumors can be a prognostic marker of several types of cancer [18]. Furthermore, immunoproteasomes represent an attractive target for the therapy of cancer [20], autoimmune [21], and neurodegenerative diseases [22,23]. Several iPs-specific inhibitors have been developed; a β5i-specific inhibitor is currently under investigation in phase II clinical trials [17,24,25,26,27].

Interestingly, the β5i subunit has been found not only in the iPs. A particular set of proteasomes containing constitutive subunits β1, β2 and β5i, or β2 and β1i, β5i has been identified in liver, kidney, intestine, colon, and the cells of other tissues irrespective of stress or inflammation [28]. These proteasomes are known as intermediate proteasomes (intPs). Little is known about the specific role of intPs. Analogously to iPs, intermediate proteasomes were shown to generate unique peptides for MHCI presentation, including those derived from cancer antigens [28,29,30]. At the same time, large amounts of intPs in different somatic cells [28] under normal conditions indicate their involvement in various aspects of cellular metabolism. Concordantly, intPs were recently demonstrated to participate in proinsulin degradation [31].

For the sake of simplicity, we will refer to iPs and intPs together as non-constitutive proteasomes (nPs).

Given the emerging role of nPs in various metabolic processes and their implication in the pathogenesis of many severe diseases including cancer, it is of high importance to develop new suitable models allowing studies of their function as well as changes in the expression of immune proteasome subunits in general and β5i as a component of both iPs and intPs in particular.

Currently, the expression of proteasome subunits is evaluated using a set of standard approaches including transcriptomic analysis, real-time PCR, immunoblotting and immunofluorescence. To study proteasome localization and interactions with regulators or other proteins, cell lines are transfected with plasmids encoding proteasome subunits fused with fluorescent proteins. Several stable cell lines expressing both constitutive and immune proteasome subunits tagged with CFP and GFP have been established [32,33,34,35]. The chimeric subunits were shown to integrate into proteasomes, allowing the characterization of proteasome localization and dynamics in living cells. At the same time, the expression of tagged proteasome subunits was not governed by endogenous gene regulation mechanisms. Therefore, changes of fluorescence intensity under various stimuli did not accurately reflect fluctuations of the endogenous subunit expression levels. Finally, chimeric subunits were likely competing with the wild-type analogues for the integration into the proteasomes.

Here, using CRISPR-Cas9n technology, we designed and characterized four human cell lines of colorectal adenocarcinoma, histiocytic lymphoma, cervix adenocarcinoma, and hepatocarcinoma origin that synthesize β5i-mCherry chimera under control of endogenous regulatory mechanisms governing PSMB8 gene expression. The cell lines were selected based on the three criteria: (i) basal level of β5i expression; (ii) the efficacy of β5i expression stimulation by the immune cytokines; and (iii) differences in origin.

## 2. Materials and Methods

### 2.1. Cell Culture

Human colorectal adenocarcinoma cell line SW620 were kindly provided by Dr. Alexey Kuzmich. The histiocytic lymphoma U937 cells were a kind gift from Prof. Carol Stocking. The cervix adenocarcinoma TZM-bl cells were generous gift from Dr. Vladimir Morozov and were originally obtained from the Centre for AIDS Reagents NIH AIDS Research and reference Reagent Program (NIH-ARP Cat# 8129-442, RRID:CVCL_B478). The hepatocarcinoma HepG2 cells were provided by Dr. Vladimir Morozov. The U937 and U937B8-mCherry cells were cultured in RPMI-1640 (Thermo Fisher Scientific, Paisley, Renfrewshire, Scotland, UK). The SW620, TZM-bl, HepG2 and SW620B8-mCherry, TZM-blB8-mCherry, HepG2B8-mCherry cells were maintained in DMEM (Thermo Fisher Scientific, Paisley, Renfrewshire, Scotland, UK). Media were supplemented with 10% fetal calf serum (FCS) (Hyclone, Logan, UT, USA), 100 U/mL penicillin and 100 μg/mL streptomycin. Cells were kept at 37 °C and 5% CO_2_.

### 2.2. Molecular Cloning

Two plasmids were obtained to perform mCherry knock-in using CRISPR-Cas9n (nickase) technology. For the first one, two gRNAs were designed to introduce nicks on the sides of the PSMB8 stop codon at a distance of 41 bp from each other and induce minimal off target cuts, the latter was verified by COSMID (https://crispr.bme.gatech.edu, accessed on 13 September 2021) and Cas-OFFinder software (http://www.rgenome.net/cas-offinder, accessed on 22 January 2020) (Table S1, Figure S1a,b). Two gRNA sequences were cloned into pDG461 vector (Addgene plasmid # 100902; http://n2t.net/addgene:100902, accessed on 13 September 2021; RRID:Addgene_100902), containing mutant Cas9(D10A) gene fused with GFP-encoding sequence (Figure S2a).

For the donor plasmid five DNA fragments (Figure S2a) were cloned simultaneously into the linearized pcDNA3.1(-) vector (Invitrogen, Waltham, MA, USA) using NEBuilder HiFi DNA Assembly Kit (New England Biolabs, Ipswich, MA, USA). The left and right homology arms with PAM blocking mutations, two adaptor sequences and sequence encoding Ser-Gly(GSGGGGSGGGGSGT) linker-mCherry chimera were obtained as PCR-products, or as synthetic sequences purchased from Evrogen, Moscow, Russia (Table S1). The assembled insert was cloned into the pAL-2T vector (Evrogen, Moscow, Russia). The pAL-2T-mCherrydonor plasmid and pDG461gRNA were amplified in E. coli cells (New England Biolabs, Ipswich, MA, USA) and were isolated using QIAprep Spin Miniprep Kit (Qiagen, Hilden, Germany), according to the manufacturer’s instructions. The quality and concentration of plasmids were assessed using a Nano Drop instrument (Thermo Scientific, Waltham, MA, USA). The integrity of inserts and the absence of mutations were confirmed by bi-directional sequencing.

### 2.3. Transfection and Cell Sorting

The SW620, TZM-bl, HepG2 and U937 cells were seeded onto 6 well plates and on the following day were co-transfected with the obtained plasmids using Lipofectamine 3000 reagent (Invitrogen, Waltham, MA, USA) according to manufacturer’s instructions. Forty-eight hours post transfection, cells were washed with PBS and all except U937 were detached from the plates with trypsin-EDTA solution (Pan-Eko, Moscow, Russia). Cells were centrifuged and resuspended in 1 mL of PBS containing 2% of FCS (HyClone, Logan, UT, USA). After that, cells with bright GFP fluorescence were sorted in a FACSAria III cell sorter (BD Biosciences, Franklin Lakes, NJ, USA). Following two weeks of propagation, obtained cells were stimulated with 1000 U/mL of recombinant human IFN-γ and 500 U/mL of recombinant human TNF-α (both from R&D systems, Minneapolis, MN, USA) for 72 h. Then, cells positive for mCherry fluorescence were sorted (Figure S2b) and after from two to five (depending on the cell line) extra cycles of propagation, induction, and sorting, the populations entirely consisting of fluorescent and inducible cells were obtained (Figures S3–S6).

### 2.4. Isolation of Genomic DNA, RNA, PCR and Real-Time PCR

Genomic DNA and total RNA were isolated using GeneJET Genomic DNA Purification Kit and GeneJET RNA Purification Kit (both from Thermo Scientific, Waltham, MA, USA), respectively, according to the manufacturer’s instructions. The concentration and purity of nucleic acids were determined using NanoDrop spectrophotometer (Thermo Scientific, Waltham, MA, USA).

**Figure 1:**
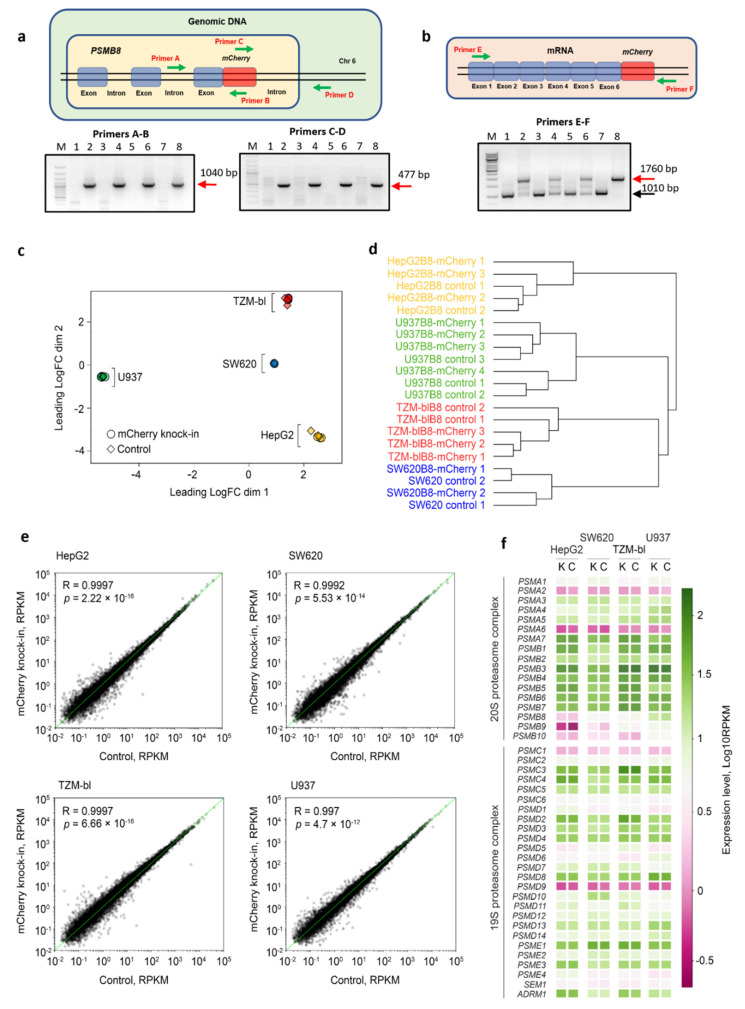
The mCherry gene is integrated into the genome and expressed in modified cells. Analysis of the gene expression in edited cells. (**a**) Gene knock-in in U937B8-mCherry, SW620B8-mCherry, TZM-blB8mCherry and HepG2B8mCherry cells was confirmed by PCR with two sets of primers using isolated genomic DNA. Following the PCR, the amplicons with expected size (1040 and 477 bp) were revealed in samples from modified cells but not control cells. Tracks are in order: (**M**) 100 bp DNA Ladder; (**1**) HepG2; (**2**) HepG2B8mCherry; (**3**) TZM-bl; (**4**) TZM-blB8mCherry; (**5**) U937; (**6**) U937B8-mCherry; (**7**) SW620; (**8**) SW620B8-mCherry. (**b**) The expression of PSMB8-mCherry chimera in modified cells was confirmed by RT-PCR. The total RNA was obtained from control and edited cells, cDNA synthesis was performed. The 1760 bp fragment represents full-length chimeric transcript, while the 1010 bp fragment corresponds to the wild-type PSMB8 transcript. Tracks are in order: (**M**) 100 bp DNA Ladder; (**1**) SW620; (**2**) SW620B8-mCherry; (**3**) HepG2; (**4**) HepG2B8mCherry; (**5**) U937; (**6**) U937B8-mCherry; (**7**) TZM-bl; (**8**) TZM-blB8mCherry. Comparative analysis of gene expression profiles of mCherry knock-in and original cell lines. (**c**) Multidimensional scaling (MDS) plot of pairwise distances calculated using root-mean-square of Log2FoldChange values between experimental groups. (**d**) Canberra distance calculated between each sample. Samples were clustered using hierarchical clustering analysis, and the dendrograms represent the clustering results. (**e**) Scatter plots demonstrating the relation of expression values (reads per kilobase per million mapped reads, RPKM) between mCherry knock-in and original cell lines. R-Pearson’s correlation coefficient, *p*-value calculated for the most variable genes (upper 75 percentile). (**f**) Heatmap depicting expression levels of the 20S and 19S proteasome complex genes (RPKM) in knock-in and original cell lines. K–mCherry knock-in cells, C–control cells.

The insertion of the transgene into genomic DNA was verified using PCR with two pairs of primers (Table S1) and Q5 High-Fidelity DNA polymerase (New England Biolabs, Ipswich, MA, USA). In the first pair, the forward primer (Primer A) was localized in the intron of PSMB8, and the reverse primer (Primer B)—in the middle of the mCherry gene (Figure 1a). In the second pair, the forward primer (Primer C) was localized in the middle of the mCherry, and the reverse primer (Primer D)—in the locus outside the PSMB8 gene (Figure 1a).

To study transgene expression the DNA was removed from RNA samples using RapidOut DNA Removal kit (Thermo Scientific, Waltham, MA, USA). The cDNA was obtained from 2 μg of total RNA using oligo(dT)20 primer and Maxima H Minus Reverse Transcriptase (Thermo Scientific, Waltham, MA, USA). A full-length chimeric transcript was amplified using primers E and F (Figure 1b, Table S1) and Q5 High-Fidelity DNA polymerase (New England Biolabs, Ipswich, MA, USA). Amplified transcripts were analyzed by bi-directional sequencing (Figure S7).

The IDT platform and the OligoAnalizer tool (Integrated DNA Technologies OligoAnalyzer, RRID:SCR_001363) were utilized to design a set of primers for the qPCR (Table S1). The forward primer (Primer G) was localized in the last exon of PSMB8 and the reverse primer (Primer H)—in the beginning of the mCherry gene to avoid a false positive signal from wild-type transcripts (Table S1). The qPCR reactions were performed as described in [36]. The β-Actin (ACTB) was used for normalization. ΔΔCt method was used to calculate the relative expression level of studied genes.

### 2.5. RNAseq

Total RNA extraction was performed from 1–1.5 × 10^6^ cells using Extract RNA reagent (Evrogen, Moscow, Russia). Each experimental group included at least two biological replicates. The concentration of RNA was measured with a Qubit Fluorometer (Invitrogen, Waltham, MA, USA). The quality of RNA was determined with an Agilent BioAnalyzer 2100 using an RNA 6000 nano kit (Agilent, Santa Clara, CA, USA). The RNA Integrity Number (RIN) of all RNA samples was not less than 8. Libraries for RNA-seq were prepared using the NEBNext Ultra II Directional RNA Library Prep Kit for Illumina (New England Biolabs, Ipswich, MA, USA), according to the manufacturer’s guidelines. Seventy-five bp single-end sequencing was conducted on an Illumina NextSeq 500 platform (Illumina, San Diego, CA, USA). Sequences reported in this study can be accessed using the GEO accession number NCBI GEO GSE183592.

Processing of raw sequence data including QC analysis, quality and adapter trimming, short read alignment (release hg38), read quantification was performed with the PPLine tool [37]. Multidimensional scaling (MDS) was conducted using the limma package [38]. RNA-sequencing count tables were statistically analyzed with the edgeR [39]. Gene set enrichment analysis (GSEA) was performed by the limma package [38] and the KEGG database [40] for all cell lines described here using a defined set of genes that show not less than 2-fold higher (for representative genes) or 2-fold lower (for non-representative genes) expression level in particular cell lines in comparison to the average expression level in all cell lines. Visualization of experimental data was made with ggplot2 and pheatmap packages [41] (https://cran.r-project.org/web/packages/pheatmap/index.html, accessed on 10 September 2021).

### 2.6. Preparation of Cellular Lysates and Western Blotting

Unstimulated and cytokine-treated cells were washed 2 times with PBS and lysed in the NP-40 cell lysis buffer (50 mM Tris-Cl (pH 8.0), 150 mM NaCl, 1.0% NP-40), left for 10 min on ice and centrifuged for 30 min at 16000× *g*. Collected supernatants were stored at −80 °C before use. Lysates were analyzed in 12% Tris-glycine polyacrylamide gels and were transferred onto nitrocellulose membranes (Bio-Rad, Hercules, CA, USA). The membranes were incubated with primary rabbit anti-β5i (Abcam, Cambridge, UK, RRID:AB_303708), or anti-mCherry (Cell Signaling, Danvers, MA, USA, RRID:AB_2799246), or anti-Rpt6 (Enzo, Farmingdale, NY, USA, RRID:AB_10555017), or anti-20S proteasome alpha1,2,3,5,6,7 (Enzo, Farmingdale, NY, USA, RRID:AB_10541045) antibodies and secondary HRP-labeled anti-rabbit or anti-mouse conjugates (Abcam, Cambridge, UK, RRID:AB_10679899 or Enzo, Farmingdale, NY, USA, RRID:AB_10540652). Blots were developed using ECLPrime kit (GE Healthcare, Little Chalfont, Buckinghamshire, UK). For signal normalization, the membranes were striped and incubated with mouse anti-β-actin antibodies (Abcam, Cambridge, UK, RRID:AB_306371) and subsequently with HRP-labeled anti-mouse antibodies (Enzo, Farmingdale, NY, USA, RRID:AB_10540652). Blots were developed as described above.

### 2.7. Immunoprecipitation of Proteasomes

Immunoprecipitation of proteasomes was performed using a Proteasome purification kit (Enzo, Farmingdale, NY, USA) according to the manufacturer’s instructions. Briefly, cells were homogenized by consecutive freezing/thawing in a buffer containing 25 mM HEPES, pH 7.4, 10% glycerol, 5 mM MgCl2, 1 mM ATP, 1 mM DTT, centrifuged for 30 min at 13,000× *g* and the supernatants were collected. Samples were incubated with the proteasome purification matrix at 4 °C overnight. After that, samples were centrifuged for 30 s 5000× *g* and the supernatants (unbound fraction) were collected. The pellet was washed 3 times in the same buffer as used for homogenization. Proteasomes were eluted using the SDS-PAGE Sample buffer (Invitrogen, Waltham, MA, USA) and analyzed by Western blotting (see above).

### 2.8. Detection of Catalytically Active Proteasome Subunits

The Me4BodipyFL-Ahx3Leu3VS (UbiQbio, Amsterdam, The Netherlands) proteasome activity probe was used to detect proteolytically active proteasome subunits using SDS-PAGE according to the described protocol [42]. Shortly, cellular lysates (app. 20 µg) were mixed with 0.5 µL of probe in PBS and were incubated for 1 h at 37 °C. Then samples were loaded into 15% Tris-Glycine polyacrylamide gel and following the electrophoresis BodipyFL fluorescence was analyzed at the excitation wavelength 480 nm and emission wavelength 530 nm using ChemiDoc XRS+ imaging system (Bio-Rad, Hercules, CA, USA). After that, the gel was stained with ROTI^®^Blue quick protein stain (Carl Roth, Karlsruhe, Germany) to ensure an equal protein load.

### 2.9. Determination of Proteasome Activity

Overall proteasome activity was determined in control and modified cells using a Me4BodipyFL-Ahx3Leu3VS (UbiQbio, Amsterdam, The Netherlands) proteasome activity probe according to the protocol for the proteasome activity measurement in fixed cells [42]. Shortly, cells were grown on the 6 well plates and 72 h after seeding, the probe was added into the culture media, reaching a final concentration of 200 nM. Cells were incubated for two hours. Adherent cells were washed with PBS and were detached from the culture plate using a trypsin solution. Suspension cells were collected and centrifuged for 3 min 250× *g* and washed with PBS. Then cells were fixed by 20 min shaking in buffer containing 1% of FBS and 0.5% formaldehyde. Detection of fluorescence intensity was performed on LSRFortessa flow cytometer (BD Biosciences, Franklin Lakes, NJ, USA).

### 2.10. Fluorescent and Confocal Microscopy

The U937, SW620, TZM-bl, HepG2 and U937B8-mCherry, SW620B8-mCherry, TZM-blB8-mCherry, HepG2B8-mCherry cells were grown on the 6 well plates (for standard fluorescent microscopy) or Clipmax culture flasks (TPP, Trasadingen, Switzerland) (for confocal microscopy of all, but U937 cell lines). Twenty-four hours after seeding cells were stimulated with 1000 U/mL of recombinant human IFN-γ (R&D systems, Minneapolis, MN, USA) and 500 U/mL of recombinant human TNF-α (R&D systems, Minneapolis, MN, USA) and incubated for additional 72 h. Cells grown for analysis using confocal microscopy were also either incubated or not incubated for 2 h with 200 nM of the Me4BodipyFL-Ahx3Leu3VS (UbiQbio, Amsterdam, The Netherlands) proteasome activity probe. After that, cells grown on 6 well plates were analyzed under Leica DMI 6000 CS or EVOS fluorescent microscopes (Leica, Wetzlar, Germany and Thermo Scientific, Waltham, MA, USA), respectively. Cells grown in Clipmax culture flasks were washed two times with PBS, fixed with 4% PFA (BosterBio, Pleasanton, CA, USA), washed with PBS and stained with NucBlue Fixed Cell ReadyProbe (Thermo Scientific, Waltham, MA, USA) for 15 min. After that, the chambers were removed and microscope slides with attached cells were covered with a SlowFade™ Gold Antifade Mountant (Thermo Scientific, Waltham, MA, USA) and cover slips (Thermo Scientific, Waltham, MA, USA). Slides were analyzed using Leica DMI 6000 CS microscope equipped with a Leica TCS SP5 laser scan unit (Leica, Wetzlar, Germany). All images were acquired in “sequential scan mode” to completely avoid a “bleed-through” effect. Areas and intensities were measured using Fiji-ImageJ software (https://imagej.net/software/fiji/, accessed on 26 August 2021, RRID:SCR_002285). Colocalization analysis was performed using Coloc2, Colocalization Threshold and JACoP plugins [43]. Image set CBS001RGM-CBS010RGM from the Colocalization Benchmark Source (www.colocalization-benchmark.com, accessed on 26 August 2021) was used to validate colocalization.

### 2.11. Flow Cytometry

For the initial assessment “wild-type” and modified cells were stimulated with IFN-γ and TNF-α (as described above). Additionally, SW620B8-mCherry cells were treated with 10, 25, 50, 100, 200, 500 or 1000 U/mL of IFN-γ, 100, 200, 500 or 1000 U/mL of TNF-α or a combination of 500 U/mL of IFN-γ and 500 U/mL of TNF-α, or 1000 U/mL of IFN-γ and 500 U/mL of TNF-α. After 72 h of incubation mCherry fluorescence was detected using LSRFortessa flow cytometer (BD Biosciences, Franklin Lakes, NJ, USA). The obtained flow cytometry data were analyzed using FlowJo version 10.0.7 (FlowJo LLC, Ashland, OR, USA; RRID:SCR_008520) and GraphPad Prism version 8.4.3. (GraphPad Software, San Diego, CA, USA; RRID:SCR_002798) software.

### 2.12. Treatment of Cells with Anti-Cancer Drugs

The SW620B8-mCherry, TZM-blB8-mCherry, HepG2B8-mCherry cells were grown on the 12 well plates and incubated for 72 h with 10 or 50 nM of Ulixertinib (Selleckchem, Houston, TX, USA), 0,5 or 5 µM of Venetoclax (Selleckchem, Houston, TX, USA), 5 or 10 µM of Ruxolitinib (Selleckchem, Houston, TX, USA), 1 or 5 nM of Vincristine (Sigma Aldrich, Saint Louis, MO, USA), 25 µM of Gefitinib (Sigma Aldrich, Saint Louis, MO, USA) and 50 or 100 nM of β5i-specific inhibitor ONX-0914 (Apexbio, Houston, TX, USA). Then fluorescence intensity was analyzed using LSRFortessa flow cytometer (BD Biosciences, Franklin Lakes, NJ, USA) as described. 

### 2.13. Statistical Analyses

Unless otherwise stated, experiments were performed at least in triplicates. Bar carts depicts mean values ± standard deviation for experimental replicates. Unpaired two-tailed *t*-test was used to evaluate the statistical significance of differences between the experimental groups. For all the experiments, *p* values less than 0.05 were regarded as statistically significant. Asterisks indicate * *p* < 0.05; ** *p* < 0.01; *** *p* < 0.001; **** *p* < 0.0001.

## 3. Results

### 3.1. The mCherry Gene Is Integrated into Genome and Expressed in Modified Cells

Modern methods of genome editing allow for precise modifications with significantly reduced number of off-target events. Here, we utilized CRISPR-Cas9n technology to perform gene knock-in and insert a gene encoding mCherry to the 3′ terminus of the ultimate (6th) exon of PSMB8 gene keeping the open reading frame intact. For this purpose, colorectal adenocarcinoma SW620, histiocytic lymphoma U937, cervix adenocarcinoma TZM-bl, and hepatocarcinoma HepG2 cells were used as backbones. The SW620 and HepG2 cells were chosen since significant expression of intPs and iPs might be expected in the colon and liver [28] and the efficient activation of β5i expression by cytokines has been already shown in SW620 cells [5]. TZM-bl cells were derived from HeLa cells—one of the most frequently used cell lines. In these cells iP subunit expression is also efficiently activated upon stimulation with cytokines. In contrast, high levels of β5i-containing proteasomes were demonstrated in the absence of cytokine stimulation in U937 cells [44], making these cells an attractive model for studying downregulation of the immunoproteasome subunit expression. 

The experiments were performed following the previously published protocol [45]. 

The work was started with the design of two expression vectors. The first was pDG461gRNA vector encoding for a Cas9-D10A (Streptococcus pyogenes)-GFP protein and two gRNAs designed to introduce 2 nicks on the sides of the PSMB8 stop codon and minimum off target cuts (Figures S1 and S2a). The second was a donor plasmid pAL2-TmCherrydonor containing an insert composed of homology arm 1, Ser-Gly linker-encoding sequence, mCherry and homology arm 2 (Figure S2a). Following co-transfection of chosen cell lines with both vectors, cells were sorted and the population with bright GFP fluorescence was isolated and propagated for two weeks. As immunoproteasome subunit expression can be efficiently induced by combined treatment with IFN-γ and TNF-α [5], transfected SW620, TZM-bl and HepG2 cells were stimulated with the cytokines and were sorted again to obtain the population of mCherry-fluorescent cells. After reiterations of incubation, stimulation, and sorting for two to five times, the SW620B8-mCherry, TZM-blB8-mCherry, HepG2B8-mCherry cell lines were obtained with 99–100% of cells with increased mCherry fluorescence following treatment with cytokines (Figures S2b, S3–S5). U937 cells were not stimulated with cytokines after transfection due to high expression of iPs and intPs [44], and two rounds of sorting and propagation were sufficient to obtain the U937B8-mCherry cell line (Figure S6).

The accuracy of transgene integration into the cellular genome was assessed using PCR with two sets of primers (Figure 1a, Table S1). Fusion of the mCherry gene to the 3′ end of the PSMB8 via a linker and conservation of the open-reading frame were confirmed by bi-directional sequencing. Furthermore, the full-length 1760 bp chimeric transcript was detected in all modified cell lines, indicating chimeric gene expression (Figure 1b). The integrity of the transcript and absence of non-synonymous nucleotide substitutions were confirmed by bi-directional sequencing (Figure S7).

### 3.2. The Control and Modified Cell Lines Demonstrate Marginal Differences in Gene Expression

As side effects of CRISPR-Cas9 genome editing could not be excluded, we performed a comparative analysis of control and modified cell transcriptomes. It was demonstrated that gene expression profiles in knock-in cells were highly similar to the original expression pattern in wild-type cells, which can be seen on multidimensional scaling and a Canberra distances dendrogram (Figure 1c,d). The Pearson correlation coefficient also shows a high similarity between knock-in and control cells ranging from R = 0.997 for U937 cells to R = 0.9997 for TZM-bl and HepG2 samples (Figure 1e). Finally, the expression of fusion β5i-mCherry did not affect the expression of 20S proteasome genes including the immune catalytic subunits PSMB8, PSMB9, and PSMB10 (fold change < 1.5, FDR > 0.1) as well as the 19S, 11Sαβ, 11Sγ and PA200 proteasome regulator genes (Figure 1f).

Given the diverse origin of cell lines used for genome editing, we performed their functional characterization (Figure S8a,b). To bring out their specificity, we selected genes showing not less than 2-fold higher or 2-fold lower expression levels in a particular cell line compared to the average expression between all four cell lines. Next, using a defined set of representative (higher expression levels) and non-representative (lower expression levels) genes, we performed the Gene set enrichment analysis using the KEGG database [40]. The analysis demonstrated that immune pathways are enriched in U937B8-mCherry cells; genes involved in metabolic pathways are present to a greater extent in HepG2B8-mCherry cells, in TZM-blB8-mCherry and SW620B8-mCherry cell lines the most representative pathways include Notch signaling, TGF-beta signaling, Rap1 signaling and Relaxin signaling (Figure S8a). The responsiveness of cells to various stimuli is associated with expressed receptor molecules. To analyze the expression of genes encoding membrane receptors, we divided genes according to different protein families including cluster of differentiation (CD) molecules, cytokine receptors, G protein-coupled receptors, and pattern recognition receptors (Figure S8b). As expected, pattern recognition receptors that play a crucial role in the innate immune response were expressed mostly in U937 cells (Figure S8b). However, the expression of the other membrane receptors including cytokine receptors, CD molecules, and G protein-coupled receptors differed among cell lines, suggesting the diverse environmental signal reception and transduction (Figure S8b).

Thus, we confirmed that genome editing, and cycles of cell propagation and sorting, had a minimal effect on general gene expression and on the expression of proteasome genes in modified cells. Moreover, predominant metabolic pathways and patterns of expressed receptors for each generated cell line were determined.

### 3.3. IFN-γ and TNF-α Stimulate Expression of β5i-mCherry Protein in Modified Cells

**Figure 2:**
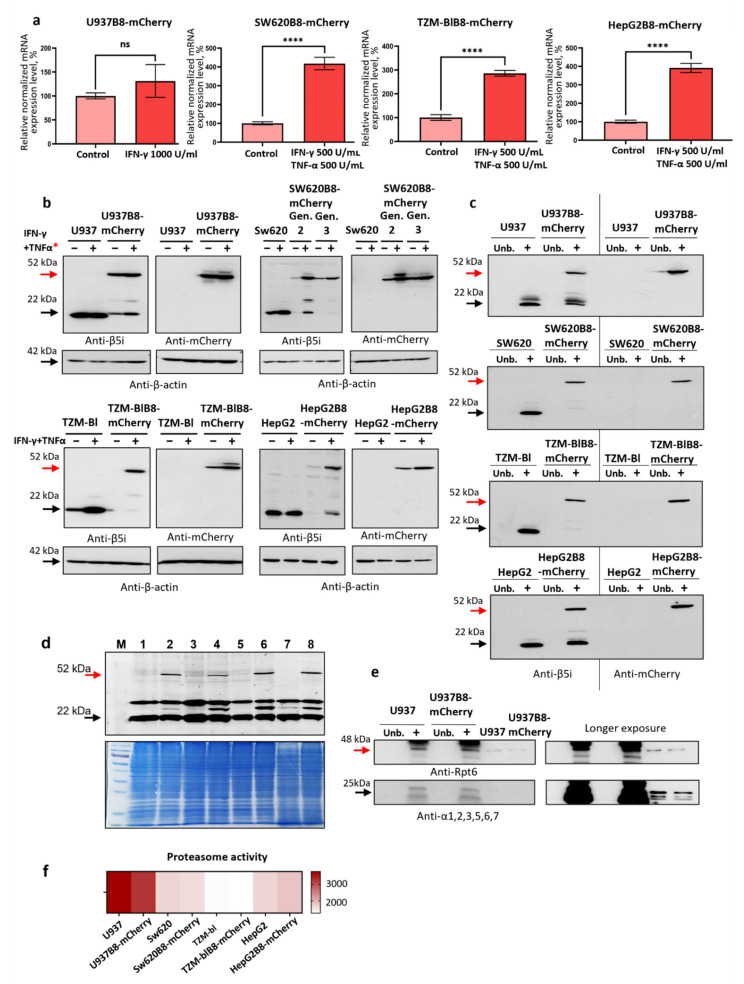
The β5i-mCherry chimera is integrated into the proteasome and is an active proteasome subunit in edited cells. (**a**) The relative expression levels of PSMB8-mCherry RNA in unstimulated U937B8-mCherry, SW620B8-mCherry, TZM-blB8mCherry and HepG2B8mCherry cells and cells treated with IFN-γ (1000 U/mL) and TNF-α (500 U/mL) for 48 h. U937B8-mCherry cells were treated with 1000 U/mL IFN-γ. Tests were performed in triplicates; n.s.—not significant, ****—*p* < 0.0001, *t*-test. (**b**) Western blot of lysates obtained from unstimulated U937, SW620, TZM-bl, HepG2, U937B8-mCherry, SW620B8-mCherry, TZM-blB8mCherry and HepG2B8mCherry cells and cells incubated with 1000 U/mL IFN-γ and 500 U/mL of TNF-α for 72 h. *—U937 and U937B8-mCherry cells were treated with 1000 U/mL IFN-γ. Lysates were run in 12% PAGE, and then proteins were transferred onto the nitrocellulose membranes. The membranes were stained either with anti-β5i or anti-mCherry primary antibodies and corresponding secondary antibodies. Blots were revealed and for the signal normalization membranes were stripped and stained with antibodies to β-actin. (**c**) Immunoprecipitation of proteasomes from lysates of IFN-γ and TNF-α- stimulated U937, SW620, TZM-bl, HepG2, U937B8-mCherry, SW620B8-mCherry, TZM-blB8mCherry and HepG2B8mCherry cells. Immunoprecipitated proteasomes were run in 12% PAGE, then proteins were transferred onto the nitrocellulose membranes. The membranes were stained either with anti-β5i or anti-mCherry primary antibodies and corresponding secondary antibodies. (**d**) β5i-mCherry is catalytically active subunit within the proteasomes in edited cells. Lysates from unstimulated control and cytokine-stimulated modified cells were incubated for 1 h at 37 °C with Me4BodipyFL-Ahx3Leu3VS proteasome activity probe. The fluorescence of proteasome subunits was analyzed in 13% Tris-Glycine polyacrylamide gel following excitation at the wavelength 480 nm and emission wavelength 530 nm (upper panel). Tracks in order: (**M**) PageRuler Prest Prot. Ladder; (**1**) SW620; (**2**) SW620B8-mCherry; (**3**) HepG2, (**4**) HepG2B8-mCherry; (**5**) TZM-bl; (**6**) TZM-blB8-mCherry; (**7**) U937; (**8**) U937B8-mCherry. Lysates of modified cells were obtained from cells treated with 1000 U/mL IFN-γ and 500 U/mL of TNF-α for 72 h. Bottom panel, the same gel was stained with Roti blue quick protein stain to ensure equal protein load. (**e**) Immunoprecipitation of proteasomes from lysates of U937 and U937B8-mCherry cells. Immunoprecipitated proteasomes were run in 12% PAGE. Proteins were transferred onto the nitrocellulose membranes. The membranes were stained with anti-19S-Rpt6 and anti-20S proteasome alpha1,2,3,5,6,7 primary mouse antibodies and corresponding secondary conjugates. (**f**) Heatmap depicting overall proteasome activity revealed in control and modified cell lines using Me4BodipyFL-Ahx3Leu3VS proteasome activity probe. Mean fluorescence intensity is shown. Detection of fluorescence intensity was performed on LSRFortessa flow cytometer (BD Biosciences, Franklin Lakes, NJ, USA).

To verify that endogenous mechanisms of PSMB8 gene expression regulation are preserved after genome editing, cDNA obtained from the modified untreated cells and cells stimulated with IFN-γ (1000 U/mL) and TNF-α (500 U/mL) was analyzed using qPCR. The two cytokines were chosen because their combination efficiently activates immunoproteasome subunit expression [5]. The stimulation of the edited cells with IFN-γ and TNF-α induced from 1.25 to a 4-fold increase in the PSMB8-mCherry gene expression, indicating that modified cells were sensitive to the cytokines and efficiently upregulated the specific mRNA synthesis upon treatment (Figure 2a). Next, we evaluated the expression of the β5i-mCherry protein. A protein with molecular weight around 52 kDa was detected by Western blotting in the lysates of SW620B8-mCherry, TZM-blB8-mCherry, HepG2B8-mCherry, and U937B8-mCherry cells (Figure 2b). The protein was detected using both antibodies to β5i and mCherry, indicating that it represents β5i-mCherry chimera (Figure 2b). Importantly, the amount of the β5i-mCherry in the cytokine-stimulated SW620B8-mCherry, TZM-blB8-mCherry, and HepG2B8-mCherry cells was significantly elevated. These data indicate that the endogenous mechanisms of β5i expression regulation are retained in the edited cells. Finally, no free mCherry was revealed in cellular lysates (Figure 2b), highlighting that almost all the mCherry molecules present in the cells are associated with the β5i proteasome subunit.

### 3.4. The β5i-mCherry Chimera Is Integrated into the Proteasome and Is an Active Proteasome Subunit in Modified Cells

Apart from the 52 kDa β5i-mCherry the 22 kDa protein corresponding to the wild-type β5i was revealed in the lysates of U937B8-mCherry and HepG2B8-mCherry cells, indicating the presence of a heterozygous cell population. At the same time, a faint band corresponding to a protein with 3–6 kDa higher molecular weight than 52 kDa was detected by Western blotting (Figure 2b). Here it should be mentioned that proteolytic proteasome subunits are synthesized as precursors and undergo posttranslational autocatalytic cleavage of propeptides during the latest stage of proteasome assembly [46]. Thus, 55–58 kDa protein (which suits the anticipated weight of full-length chimeric protein (app. 57 kDa)) might be a precursor protein, while 52 kDa protein—a subunit that underwent integration into the proteasome and subsequent cleavage of propeptide. To test this possibility, we performed immunoprecipitation of proteasome complexes from cellular lysates (Figure 2c). All modified cell lines were found to contain proteasomes with integrated β5i-mCherry (Figure 2c). To investigate whether the chimera is a catalytically active proteasome subunit, we incubated the lysates of control and cytokine-stimulated modified cells with the Me4BodipyFL-Ahx3Leu3VS proteasome activity probe. The probe covalently binds the N-terminal threonine residue of all the proteolytic subunits of the proteasome and enables their visualization [42]. Fluorescent 52 kDa protein was absent in the lysates of control cells but was readily detected in the lysates of SW620B8-mCherry, TZM-blB8-mCherry, HepG2B8-mCherry, and U937B8-mCherry cells, indicating that the chimeric subunit is a catalytically active within the proteasomes in modified cells (Figure 2d).

As the structure of mCherry might have influenced the association of proteasomes with regulators, we have carried out the Western blotting of the proteasomes immunoprecipitated from the lysates of U937 and U937B8-mCherry cells with the antibodies to the Rpt6 subunit of the 19S regulator. Almost equal amounts of Rpt6 subunits were immunoprecipitated with proteasomes from modified and wild-type cells (Figure 2e). Furthermore, using the Me4BodipyFL-Ahx3Leu3VS proteasome activity probe, we analyzed the overall proteasome activity in the control and edited cells. As a result, comparable proteasome activity was demonstrated (Figure 2f).

Taken together, we can conclude that:

(i) the chimeric subunit is integrated and is catalytically active within the proteasomes in the modified cells;
(ii) chimeric subunits are unlikely to hamper the association of 20S proteasomes with 19S regulators;
(iii) the integration of β5i-mCherry into proteasomes has minimal influence on proteasome activity in the modified cells.

### 3.5. Proteasomes with β5i-mCherry Subunit Are Localized in the Nuclei and Cytoplasm of Modified Cells

**Figure 3:**
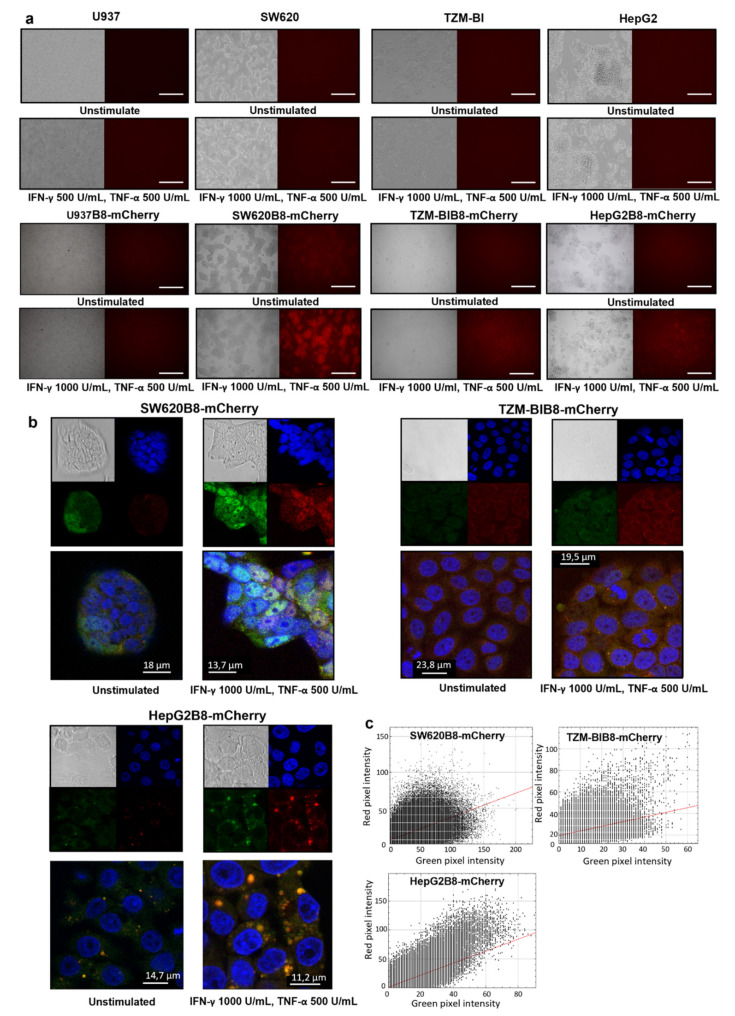
Proteasomes with β5i-mCherry subunit are localized in the nucleus and cytoplasm of modified cells. (**a**) Fluorescent microscopy of unstimulated as well as of U937, SW620, TZM-bl, HepG2, U937B8-mCherry, SW620B8-mCherry, TZM-blB8-mCherry and HepG2B8-mCherry cells treated with IFN-γ (1000 U/mL) and TNF-α (500 U/mL) for 72 h. White scale bar—400 µm. (**b**) Confocal microscopy of unstimulated and cytokine-stimulated (1000 U/mL IFN-γ and 500 U/mL TNF-α) SW620B8-mCherry, TZM-blB8-mCherry and HepG2B8-mCherry cells. mCherry fluorescence is shown in red. Additionally, cells were incubated for 2 h with Me4BodipyFL-Ahx3Leu3VS (green fluorescence) to localize proteasome activity within the cells. Cell nuclei were stained with NucBlue Fixed Cell ReadyProbe (blue fluorescence). Proteasomes were revealed in the nuclei and cytoplasm of the inspected cells. Moreover, areas with intense proteasome fluorescence were observed within the cellular cytoplasm. (**c**) Scatterplots of green (BodipyFL) and red (mCherry) pixel intensities of the modified cells treated with IFN-γ (1000 U/mL) and TNF-α (500 U/mL) for 72 h.

As the β5i-mCherry is integrated into proteasomes in SW620B8-mCherry, TZM-blB8-mCherry, HepG2B8-mCherry, and U937B8-mCherry cells, the intracellular distribution of tagged proteasomes can be investigated by fluorescent and confocal microscopy. Comparing to control or cytokine-stimulated SW620, TZM-bl or HepG2 cells (Figure 3a), a mild cytoplasmic fluorescence of mCherry was detected in SW620B8-mCherry, TZM-blB8-mCherry, and HepG2B8-mCherry cells (Figure 3a). However, following treatment with IFN-γ and TNF-α, a significant increase in cell fluorescence intensity was observed in the modified cell lines (Figure 3a). In contrast, U937B8-mCherry cells demonstrated cytoplasmic fluorescence of mCherry in the absence of cytokine stimulation, which was only moderately increased following the incubation with IFN-γ and TNF-α (Figure 3a).

To analyze the intracellular distribution of β5i-mCherry-containing proteasomes in detail, edited cells were analyzed using confocal microscopy (Figure 3b). The efficient induction of β5i-containing proteasomes following the treatment with IFN-γ and TNF-α was confirmed in SW620B8-mCherry, TZM-blB8-mCherry and HepG2B8-mCherry cells (Figure 3b and Figure S9a,b). Fluorescent proteasomes were present mostly in the cytoplasm of TZM-blB8-mCherry and HepG2B8-mCherry cells; however, an accurate analysis of slides revealed the presence of β5i-mCherry in the nucleus and the formation of spots with increased proteasome density in the cytoplasm, which was stimulated after cytokine treatment (Figure 3b). In the SW620B8-mCherry cells, the β5i-mCherry fluorescence was detected in the nucleus at a level comparable with the fluorescent signal in the cytoplasm and was significantly induced with the formation of areas with bright fluorescence following the cytokine stimulation (Figure 3b and Figure S9a,b). Moreover, the distribution of tagged proteasomes in the cytoplasm of TZM-blB8-mCherry, SW620B8-mCherry, and especially HepG2B8-mCherry cells, was not even. We observed the accumulation of proteasomes near the nucleus and around distinct cytoplasmic structures (Figure 3b and Figure S9a,b). To confirm the association of proteasome activity with β5i-mCherry-contining proteasomes in the modified cells, we analyzed the colocalization of mCherry fluorescence (red) with the fluorescence of the proteasome activity probe Me4BodipyFL-Ahx3Leu3VS (green). Indeed, red and green signals colocalized with Pearson’s colocalization coefficients (PCC) of 0.66 for HepG2B8-mCherry cell line, 0.605—for the SW620B8-mCherry cells, and 0.44—for the TZM-blB8-mCherry cells (Figure 3b,c). Furthermore, as expected, green and red fluorescence were significantly increased in all cell lines following the stimulation with cytokines.

### 3.6. Obtained Cell Lines Could Be Used for Quantitative Assessment of the Effects of Different Substances on β5i Subunit Expression

**Figure 4:**
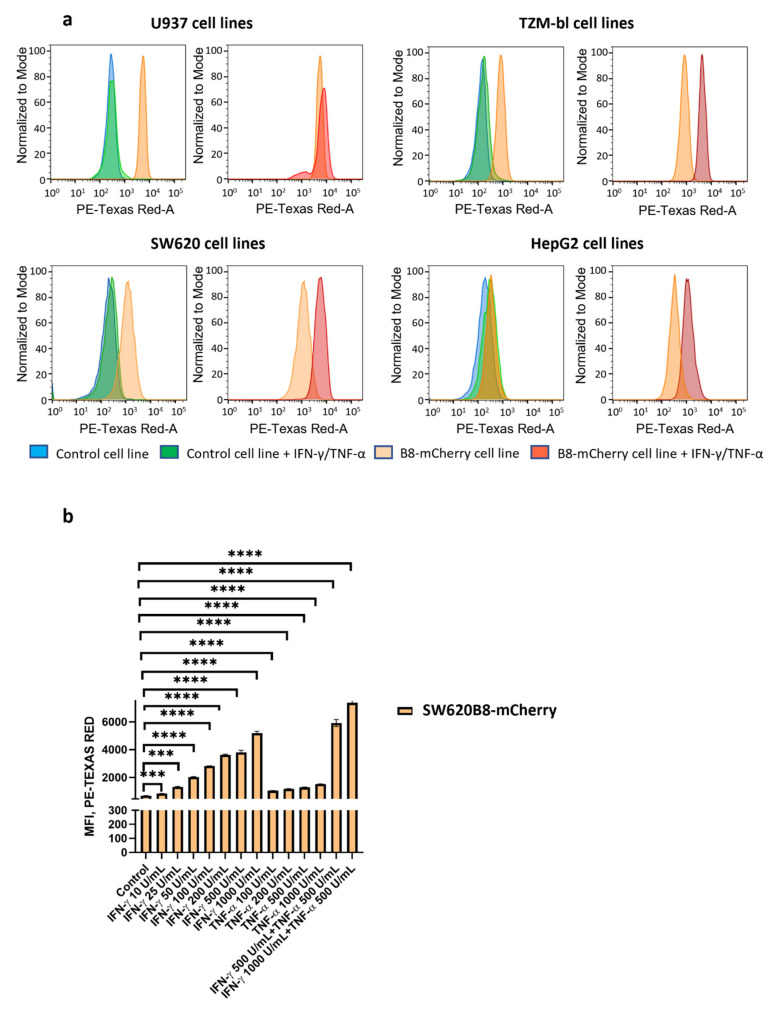
Obtained cell lines could be used for quantitative assessment of effects of different substances on the β5i subunit expression. (**a**) Flow cytometry was used for the quantitative estimation of β5i expression after stimulation of U937, SW620, TZM-bl, HepG2, U937B8-mCherry, SW620B8-mCherry, TZM-blB8-mCherry and HepG2B8-mCherry cells with 1000 U/mL of IFN-γ and 500 U/mL of TNF-α. For every pair of control and modified cell line representative histograms were obtained. Left panel—representative flow cytometry histogram showing mean cellular fluorescence of control cells (blue), cytokine-stimulated control cells (green) and unstimulated edited cells (orange). Right panel—representative flow cytometry histogram showing mean cellular fluorescence of unstimulated edited cells (orange) and edited cells treated with IFN-γ and TNF-α (red). (**b**) The fluorescence of SW620B8-mCherry cells treated with different concentrations of IFN-γ, TNF-α or two combinations of IFN-γ and TNF-α for 72 h. Tests were performed in triplicates. *** *p* < 0.001; **** *p* < 0.0001; *t*-test.

To test whether the obtained cell lines could be used for quantitative assessment of the β5i subunit expression after the stimulation with different compounds, the fluorescence of SW620, TZM-bl, HepG2, U937, SW620B8-mCherry, TZM-blB8-mCherry, HepG2B8-mCherry, and U937B8-mCherry cells following 72 h incubation with or without IFN-γ and TNF-α was evaluated by flow cytometry. Minimal differences were revealed when median fluorescence intensities (MFI) of U937, SW620, TZM-bl, HepG2 and cytokine stimulated U937, SW620, TZM-bl, HepG2, were correspondingly compared (Table 1, Figure 4a).

In contrast, U937B8-mCherry, SW620B8-mCherry, TZM-blB8-mCherry, HepG2B8-mCherry cells demonstrated significantly higher fluorescence, which was further dramatically increased as a result of IFN-γ and TNF-α treatment (Table 1, Figure 4a). To determine the sensitivity of the model system and to evaluate the effects of IFN-γ and TNF-α separately and in combination, SW620B8-mCherry cells were treated with 10, 25, 50, 100, 200, 500 or 1000 U/mL of IFN-γ, or 100, 200, 500, or 1000 U/mL of TNF-α, or 500 U/mL of IFN-γ and 500 U/mL of TNF-α, or 1000 U/mL of IFN-γ and 500 U/mL of TNF-α. A dose-dependent increase of fluorescence was observed in the SW620B8-mCherry treated with different concentrations of IFN-γ (Figure 4b). The MFI of SW620B8-mCherry cells was already increased by 21% (*p* value = 0.000019) following the incubation with 10 U/mL IFN-γ. The 100 U/mL TNF-α induced 53% (*p* value = 0.00001) increase of cellular fluorescence in SW620B8-mCherry cells. Combined treatment with 500 U/mL IFN-γ and 500 U/mL TNF-α or 1000 U/mL of IFN-γ and 500 U/mL of TNF-α dramatically increased the fluorescence of SW620B8-mCherry, implying an additive action of cytokines. These results are in line with the previously published data describing elevated β5i expression in SW620 cell line treated with both cytokines [5] (Figure 4b).

These data can serve as additional evidence that the obtained cell lines are sensitive to the same stimuli as the initial cell lines and that modified cells could be used for the quantitative assessment of β5i expression upon various treatments.

### 3.7. Anti-Cancer Drugs Ruxolitinib, Vincristine, and Gefitinib Stimulate the Expression of β5i-Containing Proteasomes in Modified Cells

**Figure 5:**
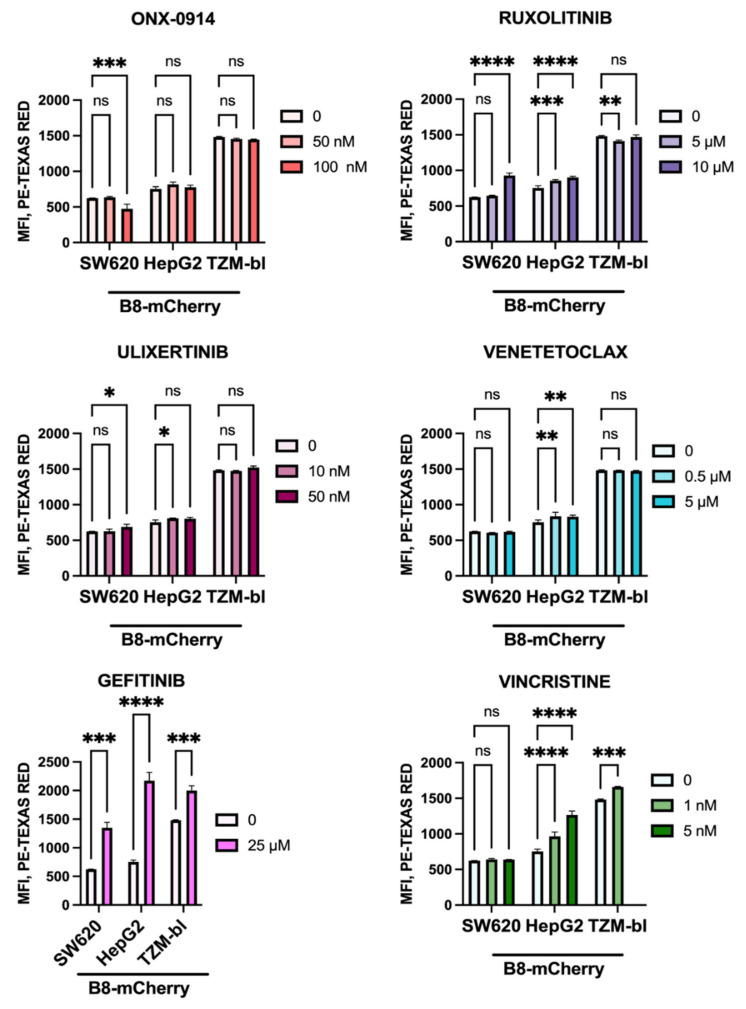
Ruxolitinib, Vincristine and Gefitinib stimulate expression of the β5i-containing proteasomes in modified cells. The SW620B8-mCherry, TZM-blB8mCherry and HepG2B8mCherry cells were treated with indicated concentrations of anti-cancer drugs Ulixertinib, Venetoclax, Ruxolitinib, Vincristine, Gefitinib and a β5i-specific inhibitor—ONX-0914 for 72 h. Beta5i-mCherry fluorescence was analyzed in treated cells by flow cytometry. Tests were performed in triplicates. * *p* < 0.05; ** *p* < 0.01; *** *p* < 0.001; **** *p* < 0.0001; *t*-test.

As a pilot practical application of obtained model cell lines, five anti-cancer drugs directed towards different targets were evaluated for their ability to stimulate intracellular β5i expression. The SW620B8-mCherry, TZM-blB8-mCherry, and HepG2B8-mCherry cells treated for 72 h with different concentrations of Ulixertinib (ERK 1 and 2 inhibitor), Venetoclax (Bcl-2 inhibitor), Ruxolitinib (JAK inhibitor), Vincristine (tubulin polymerization inhibitor), Gefitinib (EGFR inhibitor), and a β5i-specific inhibitor ONX-0914 were analyzed by flow cytometry. Vincristine had no effect on β5i expression in SW620B8-mCherry cells but upregulated β5i expression by 1,15 folds in TZM-blB8-mCherry cells (*t*-test, *** *p* < 0.001) and 1.7 folds in HepG2B8-mCherry cells (*t*-test, **** *p* < 0.0001) (Figure 5).

In contrast, Ruxolitinib stimulated chimeric protein expression more in SW620B8-mCherry cells than in HepG2B8-mCherry cells whereas it had minor effect on the TZM-blB8-mCherry cell line. Minimal effects of Ulixertinib, Venetoclax were demonstrated. At the same time, the β5i expression was increased from 2.5 to 3 folds (*t*-test, *** *p* < 0.001 or **** *p* < 0.0001) following incubation with Gefitinib in all the used cell lines (Figure 5).

## 4. Discussion

Proteasomes are responsible for the degradation of most intracellular proteins. Several forms of proteasomes with different catalytic subunit compositions of the 20S core particle have been identified. These are constitutive proteasomes with proteolytic subunits β1, β2, β5, intermediate proteasomes containing β1 or β1i, β2, β5i and immunoproteasomes with β1i, β2i and a β5i subunit set [28]. Different proteasomes seem to play, overlapping, but at the same time altering functions [10]. The non-constitutive proteasomes (intPs and iPs, together nPs) have been shown to modulate different aspects of cellular and organ homeostasis from gene expression to the immune reactions and have attracted much attention because of their role in cancer, autoimmune and neurodegenerative diseases [18]. Indeed, specific inhibitors directed against immunoproteasome subunits were developed [17,23,24,47]. The Kzr-616, a specific inhibitor of β5i, is currently under phase II clinical trials (https://www.clinicaltrials.gov/ct2/show/NCT03393013, accessed on 10 September 2021). Thus, considering the emerging role of nPs in various pathologies and the different aspects of cellular metabolism, their expression, localization, trafficking, association with other proteins, and participation in cellular contacts is of immense interest. In this regard, imaging these types of proteasomes might prove to be especially useful.

Considerable efforts have been made to visualize the proteasomes in cells. An obvious approach is the fusion of proteasome subunits with fluorescent proteins. In this regard, several important aspects should be considered: (i) The subunit expression level should not be extremely high, otherwise it would not match the expression levels of other proteasome subunits and the chimeric protein would accumulate and likely aggregate; (ii) The chimeric-subunit would compete with the endogenous counterpart during the proteasome assembly; (iii) The presence of fused fluorescent protein might hamper integration, subsequent assembly of proteasomes, or their interaction with regulators and other proteins. Several stable cell lines, or even entire organisms, expressing tagged proteasome subunits were reported [32,33,34,35,48]. Fluorescent proteins were fused with different proteasome subunits including α3, α6, α7, β7, β1i, β5i. As alpha and structural beta subunits are present in all proteasome forms, identifying a specific role for intermediate and immunoproteasomes using such models is problematic. Indeed, constitutive proteasomes are frequently prevalent in the cellular proteasome pool [10] and employing such cell lines to study nPs gets even more complicated. Therefore, only cell lines with β1i and β5i tagged subunits could be used for these purposes. Design of these cell lines significantly facilitated the progress in the field of immunoproteasome biology [32,35]. Schipper–Krom and coauthors indicate that the β5i subunit in a generated stable cell line is more efficiently integrated into proteasomes than the tagged β1i subunit, suggesting that it can stem from the strong interaction of the β5i with the chaperone (proteasome maturation protein (POMP)) [35,49]. Importantly, the authors indicate that a fluorescent protein tag likely neither hampers the association of proteasomes with regulators nor influences their catalytic activity [35]. Thus, published cell lines expressing immune proteasome subunits fused with fluorescent proteins fulfill several of the above-mentioned criteria and represent an attractive instrument to study nPs. However, till recently, stable cell lines were mostly obtained by using classical approaches based on transfection or lentiviral transduction. In this case the integration of the target gene into the genome occurs into the highly transcribed portions of euchromatin [50], leading to the high levels of transgene transcription, which is independent of the regulatory elements that govern the expression of proteasome genes.

Here, using CRISPR-Cas9n technology, we obtained a panel of cell lines expressing proteasomes with mCherry-tagged β5i. Importantly, the PSMB8-mCherry gene activation is under control of endogenous regulatory mechanisms and is efficiently induced by the “classical” stimulators of immunoproteasome gene expression, IFN-γ and TNF-α, leading to the production of fluorescent chimeric protein. We have shown that obtained cell lines could be efficiently used for quantitative assessment of the changes in the PSMB8 expression levels. Indeed, the cellular fluorescence intensity was significantly increased after the incubation with IFN-γ and TNF-α. When a particular model is used to evaluate the effect of a substance, there is always a question of sensitivity. Using flow cytometry, we determined the sensitivity of SW620B8-mCherry cells to IFN-γ and TNF-α. It was shown that the sensitivity of SW620B8-mCherry cells to IFN-γ was below 10 U/mL, which is equivalent to 0.5 ng/mL. This concentration is below the range of IFN-γ concentrations used to induce iP expression in various cells including: 8226, THP1, CCRF-CEM, HeLa, DLD-1, SW-480, BV-2, SW620, (PK)-15 cells [5,48,51,52,53]. The sensitivity of SW620B8-mCherry cells to TNF-α was below 100 U/mL. These data indicate that modified cells could be used to detect minor changes of *PSMB8* expression induced by different stimuli.

We have demonstrated that the β5i-mCherry chimera efficiently integrates into proteasomes and represents a catalytically active subunit within. Thus, we studied the intracellular distribution of proteasomes in SW620B8-mCherry, TZM-blB8-mCherry, HepG2B8-mCherry and U937B8-mCherry cells and showed nuclear and cytoplasmic localization of β5i-containing proteasomes in adherent cells. These data are in agreement with the previously published results [54]. Interestingly, the distribution of proteasomes in the cytoplasm was not equal; the fluorescent signal was stronger around nucleus encapsulating a large cytoplasmic structure. This structure might represent the endoplasmic reticulum, corroborating previous results obtained by immunofluorescence with monoclonal antibodies to the proteasome immune subunits [54]. Furthermore, after the stimulation of cells with cytokines, we detected the formation of cytoplasmic aggregates enriched with proteasomes, which was most significant in HepG2B8-mCherry cells. The aggregates with a high proteasome activity were present in control HepG2 cells stimulated with IFN-γ and TNF-α as well, indicating that their formation was not associated with the presence of mCherry fused with β5i (Figure S10). The nature of these aggregates should be specifically addressed; however, IFN-γ and TNF-α were shown to induce the formation of stress granules [55]. The proteasomes are in turn recruited to the stress granules to promote their clearance [56]. Alternatively, proteasome accumulation into the proteasome clusters, storage granules or resembling structures could not be ruled out [57,58]. It should be mentioned that antibodies that are used for the immunofluorescent detection of immunoproteasomes might have a certain background due to unspecific reaction with different cellular proteins. Our cells lack this drawback, since the unbound mCherry was not observed within the modified cells (Figure 2b).

The elevated immunoproteasome gene expression is associated with better prognosis for several types of cancer (https://www.proteinatlas.org/ENSG00000204264-PSMB8/pathology, accessed on 12 July 2021). Therefore, we studied the effects of current and perspective anti-cancer drugs on β5i expression. Indeed, β5i expression was stimulated in TZM-blB8-mCherry and HepG2B8-mCherry cells after the treatment with Vincristine. Ruxolitinib mostly affected β5i expression in SW620B8-mCherry cells. Such discrepancies are likely associated with the differences between cell lines, the patterns of expressed membrane molecules and prevailing signaling pathways (Figure S8). Interestingly, the highest activation level of β5i expression was found in all edited cell lines following the incubation with the EGFR inhibitor Gefitinib. Transcriptomic studies revealed an absence of EGFR expression in the modified cells except TZM-blB8mCherry (Figure S11), indicating an alternative mode of Gefitinib action. Indeed, Gefitinib was demonstrated to indirectly induce oxidative stress in various cell lines [59,60,61]. Induction of oxidative stress in turn stimulates immunoproteasome synthesis with the possible involvement of the IRF-1 signal transduction pathway, the cAMP/cGMP pathway, and the NF-κB pathway [62,63]. Thus, the activation of immunoproteasome gene expression might be caused by Gefitinib-induced oxidative stress. Furthermore, Gefitinib was shown to have several off targets, including various tyrosine kinases [64,65]. Therefore, we analyzed the expression of 23 genes encoding Gefitinib off-targets in the obtained cell lines (Figure S11). Among those, 6 were not expressed, or they demonstrated extremely low expression levels, while 13 demonstrated significant expression levels of in all cell lines. Moreover, KEGG pathway analysis [66], performed using a MetaboSignal R package [67], revealed metabolic relationships between these proteins and transcription factors involved in PSMB8 regulation described in [68] including NF-κB, IRF-1 and CREB. Consequently, β5i activation might be associated with the modulation of the activity of one or several of those proteins by Gefitinib. Although precise molecular mechanisms underlying the activation of β5i remain obscure and should be addressed in further studies, the obtained data highlight the unexpected side effects of Gefitinib and represent the first evidence of nPs stimulation by Gefitinib. Activation of nPs expression broadens the repertoire of peptides generated in cells and presented on the cellular membrane in complexes with MHC I molecules [29,69,70]. This can make cancer cells more “visible” to the immune system. Therefore, the modulation of immunoproteasome expression by certain anti-cancer drugs might be considered as an additional unexpected molecular mechanism, supporting the beneficiary action of these compounds in cancer therapy.

## 5. Conclusions

Taken together, obtained cell lines provide not only fast and convenient tools to measure the effect of practically any compound on the β5i expression, but allows studying non-constitutive proteasome localization, trafficking, interaction with other proteins, formation of complexes, transformation of the proteasome pool following oxidative stress, administration of toxins, viral and bacterial infections, temperature fluctuations, intercellular communication via delivery of proteasomes by extracellular vesicles, and various other aspects of nP molecular biology in real time and in living cells. Finally, the obtained data enables the selection of a particular cell line to study the effects of a given drug or condition on β5i proteasome subunit expression based on the cell line origin, activated pathways and receptor expression profiles.

## Figures and Tables

**Table 1:** Relative median fluorescent intensity of 10.000 control and modified cells following 72 h incubation with 1000 U/mL of IFN-γ and 500 U/mL of TNF-α. Three independent repeats were performed for each cell line. The standard deviation is below 10% in each case. The MFI of control untreated cells is 100%.

(Cytokine Stimulation)	Cell Lines							
Relative MFI, %	SW620	SW620B8-mCherry	TZM-bl	TZM-blB8-mCherry	HepG2	HepG2B8-mCherry	U937	U937B8-mCherry
No	100	581	100	610	100	200	100	1928
Yes	123	2778	124	3199	158	626	109	2460

## Data Availability

Cell lines generated in this study will be made available on request, but we may require a payment and/or a completed Materials Transfer Agreement if there is potential for commercial application. Sequences reported in this study can be accessed using the GEO accession number NCBI GEO GSE183592. Other data generated during and/or analyzed during the current study are available from the corresponding author on reasonable request.

## Supplementary Materials

The following are available online at https://www.mdpi.com/article/10.3390/cells10113049/s1, Figure S1: gRNA design and identification of putative off target cleavage sites, Figure S2: Vector design and experimental pipeline, Figure S3: Gating strategy for the sorting of SW620B8-mCherry cells, Figure S4: Gating strategy for the sorting of TZM-blB8-mCherry cells, Figure S5: Gating strategy for the sorting of HepG2B8-mCherry cells, Figure S6: Gating strategy for the sorting of U937B8-mCherry cells, Figure S7: Multiple alignment of PSMB8-mCherry chimera transcript sequences from the obtained cell lines, Figure S8: Comparative functional analysis of knock-in cells, Figure S9: Confocal microscopy of the unstimulated and SW620B8-mCherry cells treated with 1000 U/mL IFN-γ and 500 U/mL of TNF-α, Figure S10: Confocal microscopy of the HepG2 cells stimulated with IFN-γ (1000 U/mL) and TNF-α (500 U/mL), Figure S11: The expression of EGFR gene and 23 Gefitinib off target genes in the modified cells, Table S1: Primers used in the study.
