# Supplementary material for: A Cell-Based Platform for the Investigation of Immunoproteasome Subunit β5i Expression and Biology of β5i-Containing Proteasomes
Source: Cells. 2021 Nov 5;10(11):3049. doi: 10.3390/cells10113049 (PMC8616536; doi:10.3390/cells10113049)
Supplement: Supplementary file 1 [file cells-10-03049-s001.zip › Supplementary materials - Figures.pdf]

a

gRNA_1

Bulge Type	Target	Chromosome	Position	Direction	Mismatches	Bulge Size
X	crRNA: TGGCTTCCCGGTACTGGTGCAGCNGG<br>DNA: TGGCTTCCCGGTACTGGTGCAGCAGG	chr6	32840966	+	0	0
DNA	crRNA: T-GGCTTCCCGGTACTGGTGCAGCNGG<br>DNA: TTGGCTTCCCGGTACTGGTGCAGCAGG	chr6	32840965	+	0	1
DNA	crRNA: TG-GCTTCCCGGTACTGGTGCAGCNGG<br>DNA: TtGGCTTCCCGGTACTGGTGCAGCAGG	chr6	32840965	+	1	1
DNA	crRNA: TGG-CTTCCCGGTACTGGTGCAGCNGG<br>DNA: TtGGCTTCCCGGTACTGGTGCAGCAGG	chr6	32840965	+	1	1
DNA	crRNA: TGGC-TTCCCGGTACTGGTGCAGCNGG<br>DNA: TtGgCTTCCCGGTACTGGTGCAGCAGG	chr6	32840965	+	2	1
RNA	crRNA: TGGCTTCCCGGTACTGGTGCAGCNGG<br>DNA: g-GCTTCCCGGTACTGGTGCAGCAGG	chr6	32840967	+	1	1
RNA	crRNA: TGGCTTCCCGGTACTGGTGCAGCNGG<br>DNA: gG-CTTCCCGGTACTGGTGCAGCAGG	chr6	32840967	+	1	1
RNA	crRNA: TGGCTTCCCGGTACTGGTGCAGCNGG<br>DNA: gGc-TTCCCGGTACTGGTGCAGCAGG	chr6	32840967	+	2	1

gRNA_2

Bulge Type	Target	Chromosome	Position	Direction	Mismatches	Bulge Size
RNA	crRNA: TCAATAATGGTGGTGGTGGCAGCGTNGG<br>DNA: TCAATAATGGTGGTGGTGGCA-gcTGGG	chr6	32840938	-	2	1
RNA	crRNA: TCAATAATGGTGGTGGTGGCAGCGTNGG<br>DNA: TCAATAATGGTGGTGGTGGCAG-cTGGG	chr6	32840938	-	1	1
RNA	crRNA: TCAATAATGGTGGTGGTGGCAGCGTNGG<br>DNA: TCAATAATGGTGGTGGTGGCAGC-TGGG	chr6	32840938	-	0	1

b

gRNA_1

Result	Query type	Mismatch	Hit ends in RG	chr position	Strand	Cut site	Score
TGGCTTCCCGGTACTGGTGCAGCAGG -- hit<br>TGGCTTCCCGGTACTGGTGCAGCNGG -- query	No indel	0	Yes	Chr6:32840967-32840992	+	32840986	0
TGGCTGCCGGGCACTGGTGCAGCAGG -- hit<br>TGGCTTCCCGGTACTGGTGCAGCNGG -- query	No indel	3	Yes	Chr1:114152590-114152615	+	114152609	0.71
^GGCTTCCCGGTACTGGTGCAGCAGG -- hit<br>TGGCTTCCCGGTACTGGTGCAGCNGG -- query	Del 23	0	Yes	Chr6:32840968-32840992	+	32840986	0.61
G^GCTTCCCGGTACTGGTGCAGCAGG -- hit<br>TGGCTTCCCGGTACTGGTGCAGCNGG -- query	Del 21, or Del 22	1	Yes	Chr6:32840968-32840992	+	32840986	0.71
GGC^TTCCCGGTACTGGTGCAGCAGG -- hit<br>TGGCTTCCCGGTACTGGTGCAGCNGG -- query	Del 20	2	Yes	Chr6:32840968-32840992	+	32840986	0.83
TGGCTTCCCGGTACTGGTGCA^GCAG -- hit<br>TGGCTTCCCGGTACTGGTGCAGCNGG -- query	Del 2	2	Yes	Chr6:32840967-32840991	+	32840985	31.51
TGGCTTCCCGGTACTGGTGCAG^CAG -- hit<br>TGGCTTCCCGGTACTGGTGCAGCNGG -- query	Del 1	1	Yes	Chr6:32840967-32840991	+	32840985	26.51
TGGCTTCCCGGTACTGGTGCAGC^AG -- hit<br>TGGCTTCCCGGTACTGGTGCAGCNGG -- query	Del PAM 3	1	Yes	Chr6:32840967-32840991	+	32840985	40.51
TGGCTTCCCGGTACTGGTGCAGCA^G -- hit<br>TGGCTTCCCGGTACTGGTGCAGCNGG -- query	Del PAM 1, or Del PAM 2	0	Yes	Chr6:32840967-32840991	+	32840985	20.51
TGGCTTCCCGGTACTGGTGCAGC^AG -- hit<br>TGGCTTCCCGGTACTGGTGCAGCNGG -- query	Del PAM 3	1	Yes	Chr6:32840967-32840991	+	32840985	40.51
TGGCTTCCCGGTACTGGTGCAGCA^G -- hit<br>TGGCTTCCCGGTACTGGTGCAGCNGG -- query	Del PAM 1, or Del PAM 2	0	Yes	Chr6:32840967-32840991	+	32840985	20.51
TTGGCTTCCCGGTACTGGTGCAGCAGG -- hit<br>TNGGCTTCCCGGTACTGGTGCAGCNGG -- query	Ins 22	0	Yes	Chr6:32840966-32840992	+	32840986	0.8
TTGGCTTCCCGGTACTGGTGCAGCAGG -- hit<br>TGNGCTTCCCGGTACTGGTGCAGCNGG -- query	Ins 21	1	Yes	Chr6:32840966-32840992	+	32840986	0.9
TTGGCTTCCCGGTACTGGTGCAGCAGG -- hit<br>TGGNCTTCCCGGTACTGGTGCAGCNGG -- query	Ins 20	1	Yes	Chr6:32840966-32840992	+	32840986	0.92
TTGGCTTCCCGGTACTGGTGCAGCAGG -- hit<br>TGGCNTTCCCGGTACTGGTGCAGCNGG -- query	Ins 19	2	Yes	Chr6:32840966-32840992	+	32840986	1.05
TGGCTTCCCGGTACTGGTGCAGCAGGT -- hit<br>TGGCTTCCCGGTACTGGTGCAGNCNGG -- query	Ins 1	2	No	Chr6:32840967-32840993	+	32840987	32.7
TGGCTTCCCGGTACTGGTGCAGCAGGT -- hit<br>TGGCTTCCCGGTACTGGTGCAGCNNGG -- query	Ins PAM 2, or Ins PAM 3	1	No	Chr6:32840967-32840993	+	32840987	40.7
TGGCTTCCCGGTACTGGTGCAGCAGGT -- hit<br>TGGCTTCCCGGTACTGGTGCAGCNGNG -- query	Ins PAM 1	1	No	Chr6:32840967-32840993	+	32840987	40.7

gRNA_2

Result	Query type	Mismatch	Hit ends in RG	chr position	Strand	Cut site	Score
TCAATAATGGTGGTGGTGGCAGCTGGGC -- hit<br>TCAATAATGGTGGTGGTGGCAGCGTNGG -- query	No indel	3	No	Chr6:32840938-32840965	-	32840944	31
TCAATAATGGTGGTGGTGGCA^GCTGGG -- hit<br>TCAATAATGGTGGTGGTGGCAGCGTNGG -- query	Del 4	2	Yes	Chr6:32840939-32840965	-	32840945	12.51
TCAATAATGGTGGTGGTGGCAG^CTGGG -- hit<br>TCAATAATGGTGGTGGTGGCAGCGTNGG -- query	Del 3	1	Yes	Chr6:32840939-32840965	-	32840945	9.51
TCAATAATGGTGGTGGTGGCAGC^TGGG -- hit<br>TCAATAATGGTGGTGGTGGCAGCGTNGG -- query	Del 2	0	Yes	Chr6:32840939-32840965	-	32840945	5.51
TCAATAATGGTGGTGGTGGCAGCT^GGG -- hit<br>TCAATAATGGTGGTGGTGGCAGCGTNGG -- query	Del 1	1	Yes	Chr6:32840939-32840965	-	32840945	11.51
TCAATAATGGTGGTGGTGGCAGCTG^GG -- hit<br>TCAATAATGGTGGTGGTGGCAGCGTNGG -- query	Del PAM 3	2	Yes	Chr6:32840939-32840965	-	32840945	31.51
TCAATAATGGTGGTGGTGGCAGCTGG^G -- hit<br>TCAATAATGGTGGTGGTGGCAGCGTNGG -- query	Del PAM 1, or Del PAM 2	2	Yes	Chr6:32840939-32840965	-	32840945	31.51

Supplementary Figure S1. gRNA design and identification of putative off target cleavage sites. (**a**) Identification of possible gRNA off target sites with COSMID (https://crispr.bme.gatech.edu/) and (**b**) Cas-OFFinder software (http://www.rgenome.net/cas-offinder/).

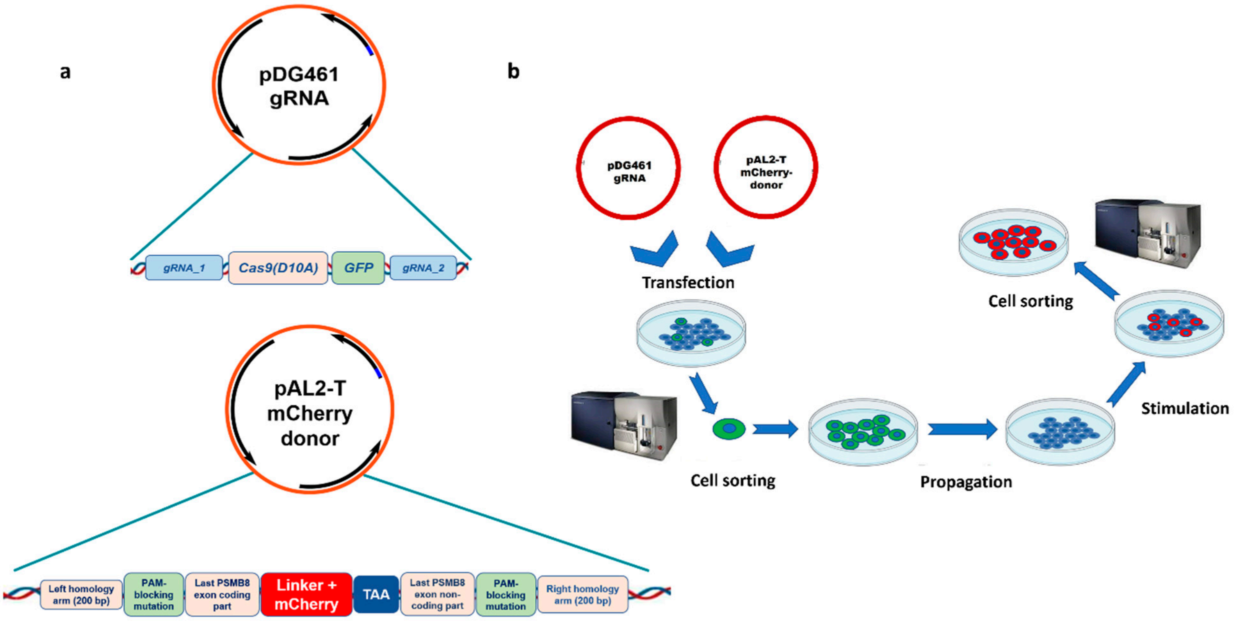

Supplementary Figure S2. Vector design and experimental pipeline. (**a**) Schematic representation of pDG461gRNA and pAL-2TmCherrydonor plasmids. The obtained pDG461gRNA vector encodes the Cas9D10A mutant endonuclease, green fluorescent protein (GFP) and contains sequences of two gRNAs designed to introduce 2 nicks on the sides of the *PSMB8* stop codon at a distance of 41 bp from each other. The commercial vector pAL-2T was used as a backbone for pAL-2TmCherrydonor plasmid. Gibson assembly method was used to obtain an insert composed of 5′ homology arm, fragment of the *PSMB8* gene, Ser-Gly linker encoding sequence, *mCherry* and the 3′ homology arm. (**b**) Schematic representation of experiments performed with U937, SW620, TZM-bl and HepG2 cells during genome editing, selection and enrichment of cellular population with modified genome. Cell lines were co-transfected with obtained plasmids. Forty-eight h post transfection cell sorting was performed and cells with high GFP expression were obtained. After two weeks of subsequent cell propagation GFP fluorescence was significantly reduced, and cells were incubated with 1000 U/mL of IFN-γ and 500 U/mL of TNF-α for 72 h and analyzed for mCherry fluorescence. Cells were sorted again to enrich the population of cells with mCherry fluorescence.

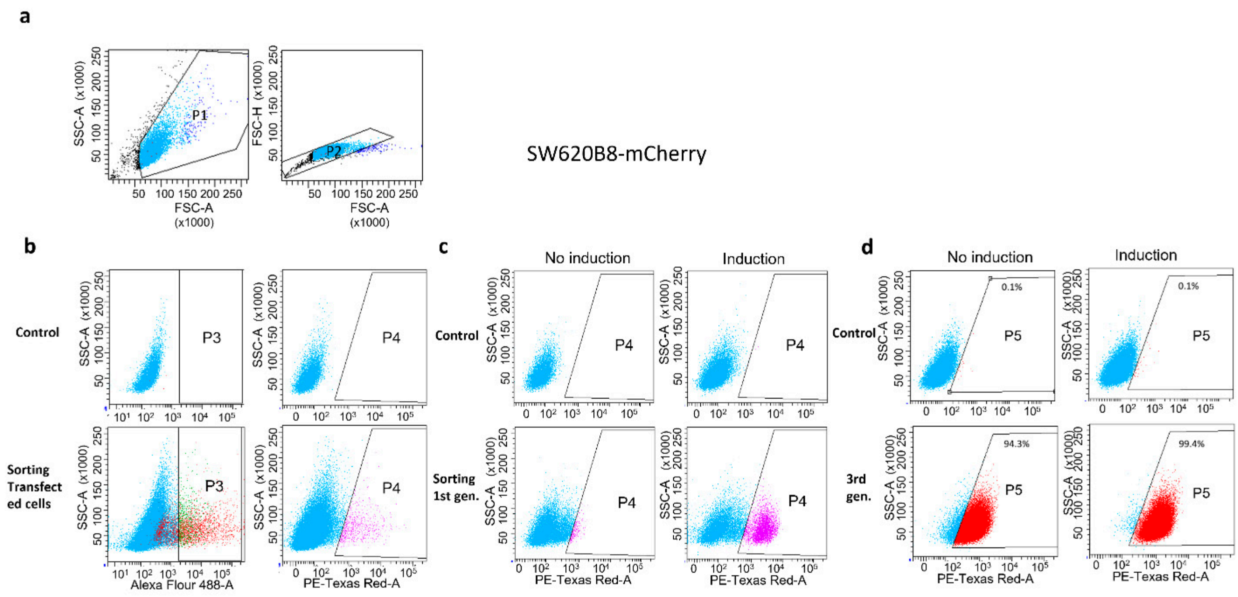

Supplementary Figure S3. Gating strategy for the sorting of SW620B8-mCherry cells. (**a**) FSC vs SSC gating to exclude dead cells and cell debris, FSC area vs height gating to exclude cell doublets. (**b**) After transfection, GFP+ (gate P3) and mCherry+ (gate P4) cell population was isolated by FACS. (**c**) mCherry+ SW620 cells were sorted after induction with 1000 U/mL of IFN-γ and 500 U/mL of TNF-α for 72 h by FACS (gate P4). (**d**) The percentage of mCherry+ cells (gate P5) after 3 rounds of propagation, induction, and sorting. Control – wild-type cells.

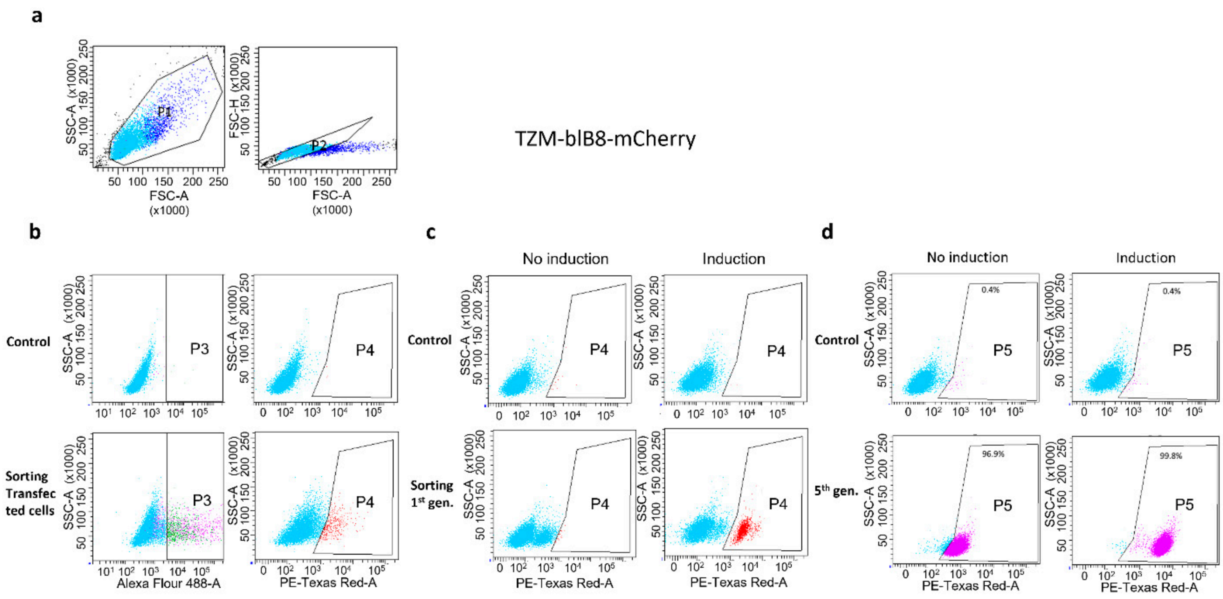

Supplementary Figure S4. Gating strategy for the sorting of TZM-blB8-mCherry cells. (**a**) FSC vs SSC gating to exclude dead cells and cell debris, FSC area vs height gating to exclude cell doublets. (**b**) After transfection, GFP+ (gate P3) and mCherry+ (gate P4) cell population was isolated by FACS. (**c**) mCherry+ TZM-bl cells were sorted after induction with 1000 U/mL of IFN-γ and 500 U/mL of TNF-α for 72 h by FACS (gate P4). (**d**) The percentage of mCherry+ cells (gate P5) after 5 rounds of propagation, induction, and sorting. Control – wild-type cells.

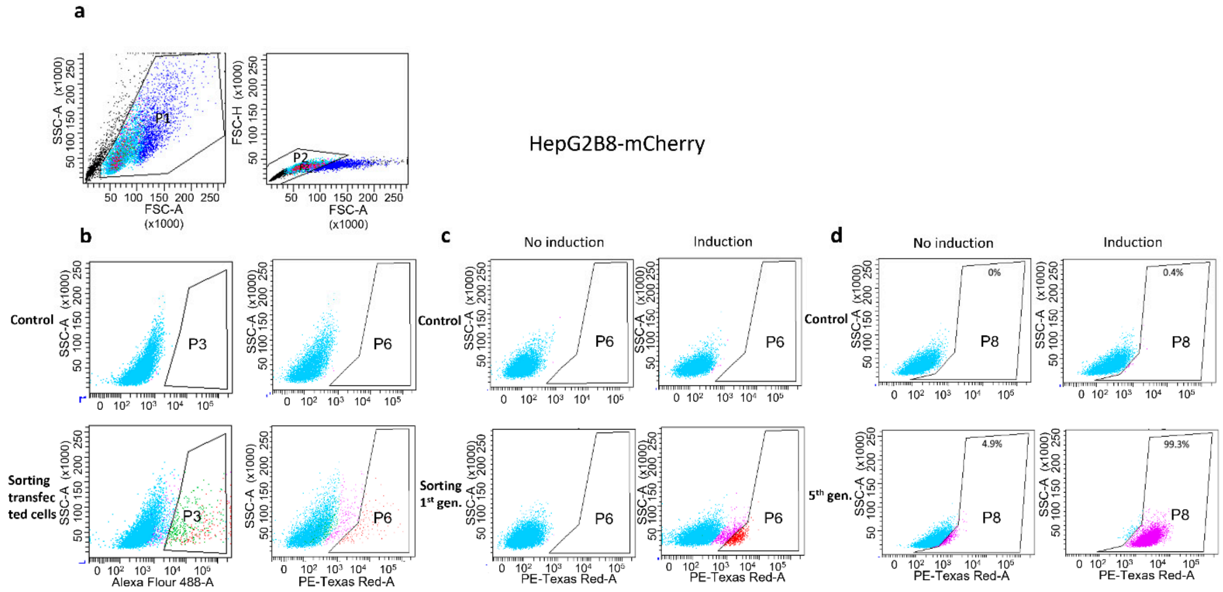

Supplementary Figure S5. Gating strategy for the sorting of HepG2B8-mCherry cells. (**a**) FSC vs SSC gating to exclude dead cells and cell debris, FSC area vs height gating to exclude cell doublets. (**b**) After transfection, GFP+ (gate P3) and mCherry+ (gate P4) cell population was isolated by FACS. (**c**) mCherry+ HepG2 cells were sorted after induction with 1000 U/mL of IFN-γ and 500 U/mL of TNF-α for 72 h by FACS (gate P4). (**d**) The percentage of mCherry+ cells (gate P5) after 5 rounds of propagation, induction, and sorting. Control – wild-type cells.

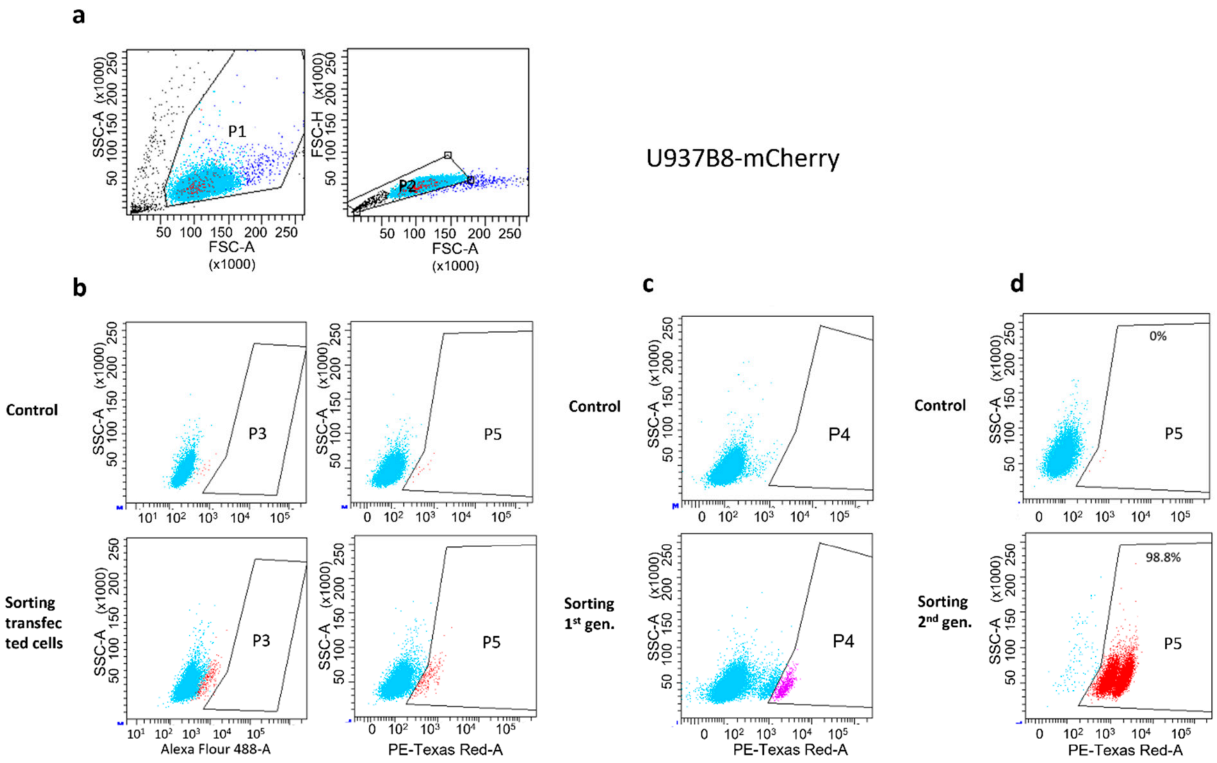

Supplementary Figure S6. Gating strategy for the sorting of U937B8-mCherry cells. (**a**) FSC vs SSC gating to exclude dead cells and cell debris, FSC area vs height gating to exclude cell doublets. (**b**) After transfection, GFP+ (gate P3) and mCherry+ (gate P4) cell population was isolated by FACS. (**c**) mCherry+ U937 cells were sorted after induction with 1000 U/mL of IFN-γ and 500 U/mL of TNF-α for 72 h by FACS (gate P4). (**d**) The percentage of mCherry+ cells (gate P5) after 2 rounds of propagation, induction, and sorting. Control – wild-type cells.

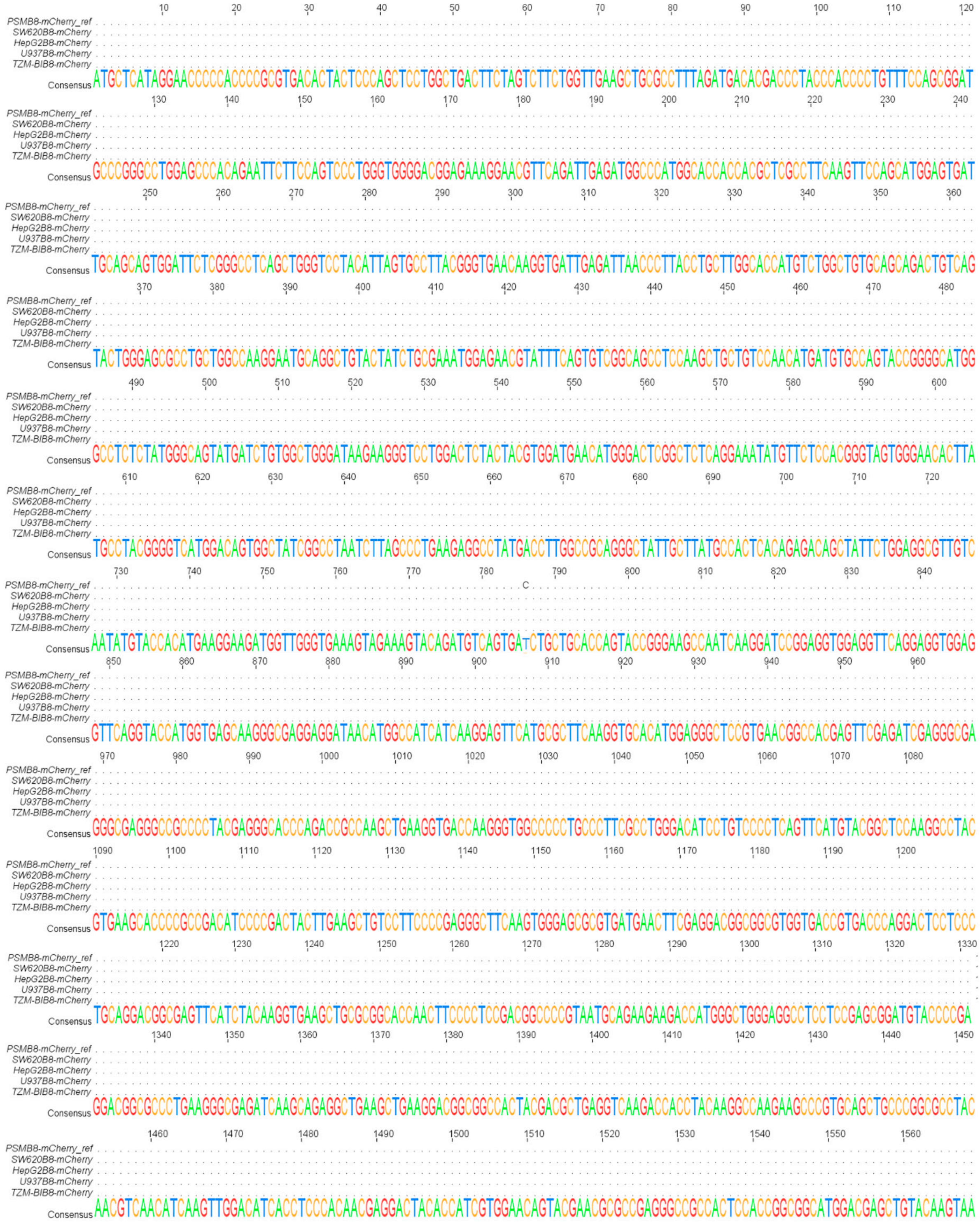

Supplementary Figure S7. Multiple alignment of PSMB8-mCherry chimera transcript sequences from the obtained cell lines. Multiple alignment of PSMB8-mCherry chimera transcript sequences amplified from U937B8-mCherry, SW620B8-mCherry, TZM-blB8mCherry and HepG2B8mCherry cells. *PSMB8* transcript sequence NM_004159.5 was used as a reference.

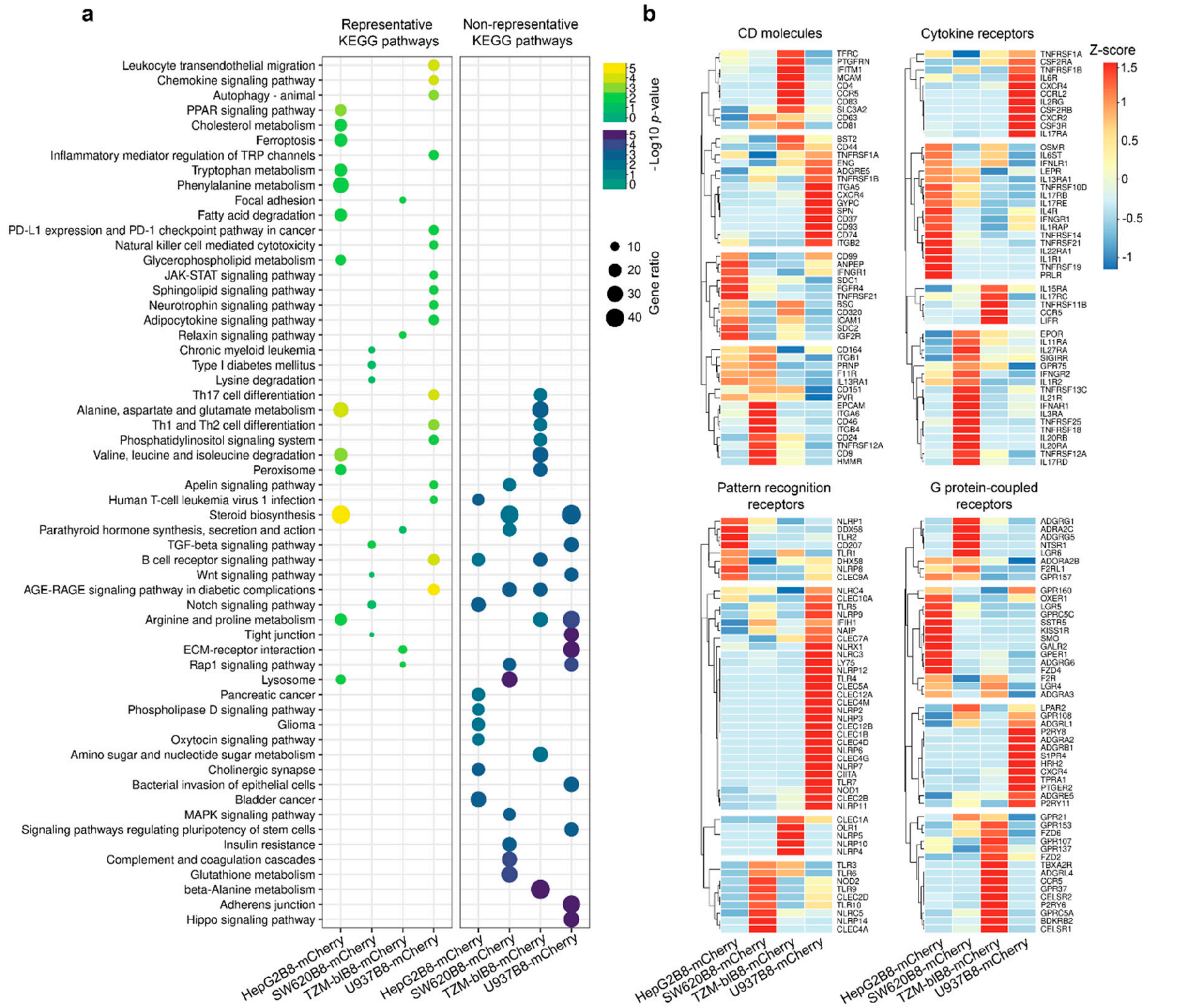

Supplementary Figure S8. Comparative functional analysis of knock-in cells. (**a**) Gene set enrichment analysis (GSEA) in the KEGG database depicting representative and non-representative pathways in each cell type. Only genes that show not less than the 2-fold difference (higher for representative; lower for non-representative pathways) in expression level (reads per kilobase per million mapped reads, RPKM) in one cell type in comparison to the average expression level in all cell types were used for enrichment analysis. Only gene expression values in mCherry knock-in cells were used in this analysis. P-values for enrichment analysis are shown using different color keys for representative and non-representative pathways. Gene ratio shows the percentage of representative or non-representative genes from the overall number of genes in the pathway. (**b**) Heatmaps demonstrating expression levels of top 50 receptors based on expression values from different protein families. Expression values (RPKM) are Z-transformed.

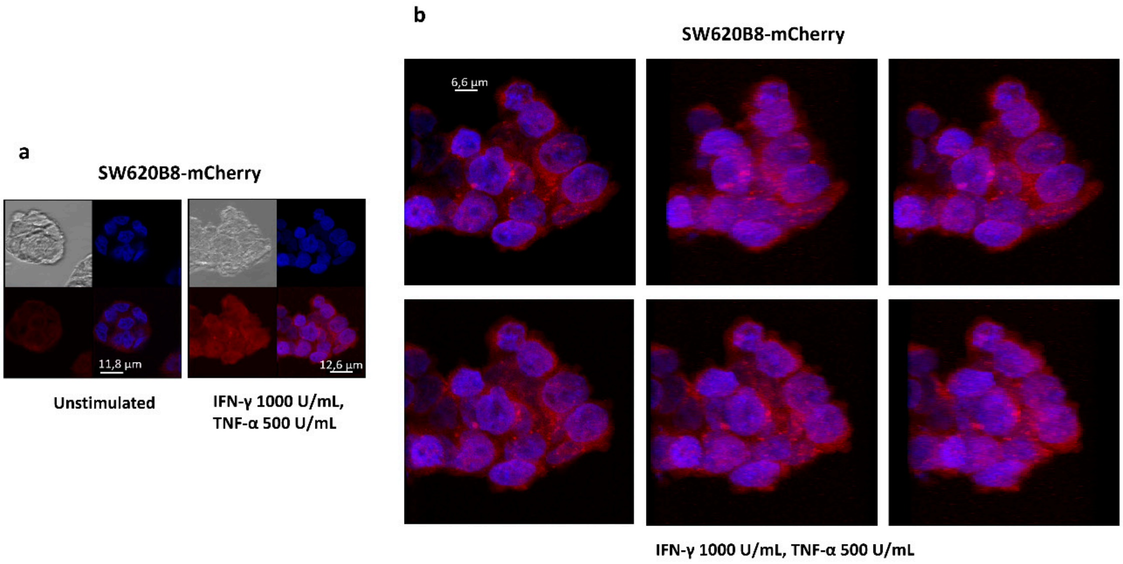

Supplementary Figure S9. Confocal microscopy of the unstimulated and SW620B8-mCherry cells treated with 1000 U/mL IFN-γ and 500 U/mL of TNF-α. (**a**) Confocal microscopy of the unstimulated and SW620B8-mCherry cells treated with 1000 U/mL IFN-γ and 500 U/mL of TNF-α. (**b**) Images of stimulated cells shown in **a** under different angles.

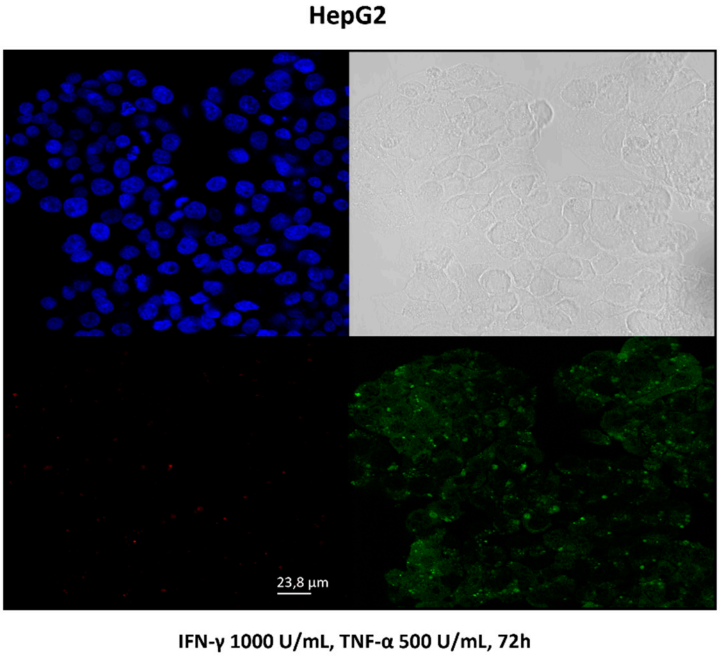

Supplementary Figure S10. Confocal microscopy of the HepG2 cells stimulated with IFN-γ (1000 U/mL) and TNF-α (500 U/mL).

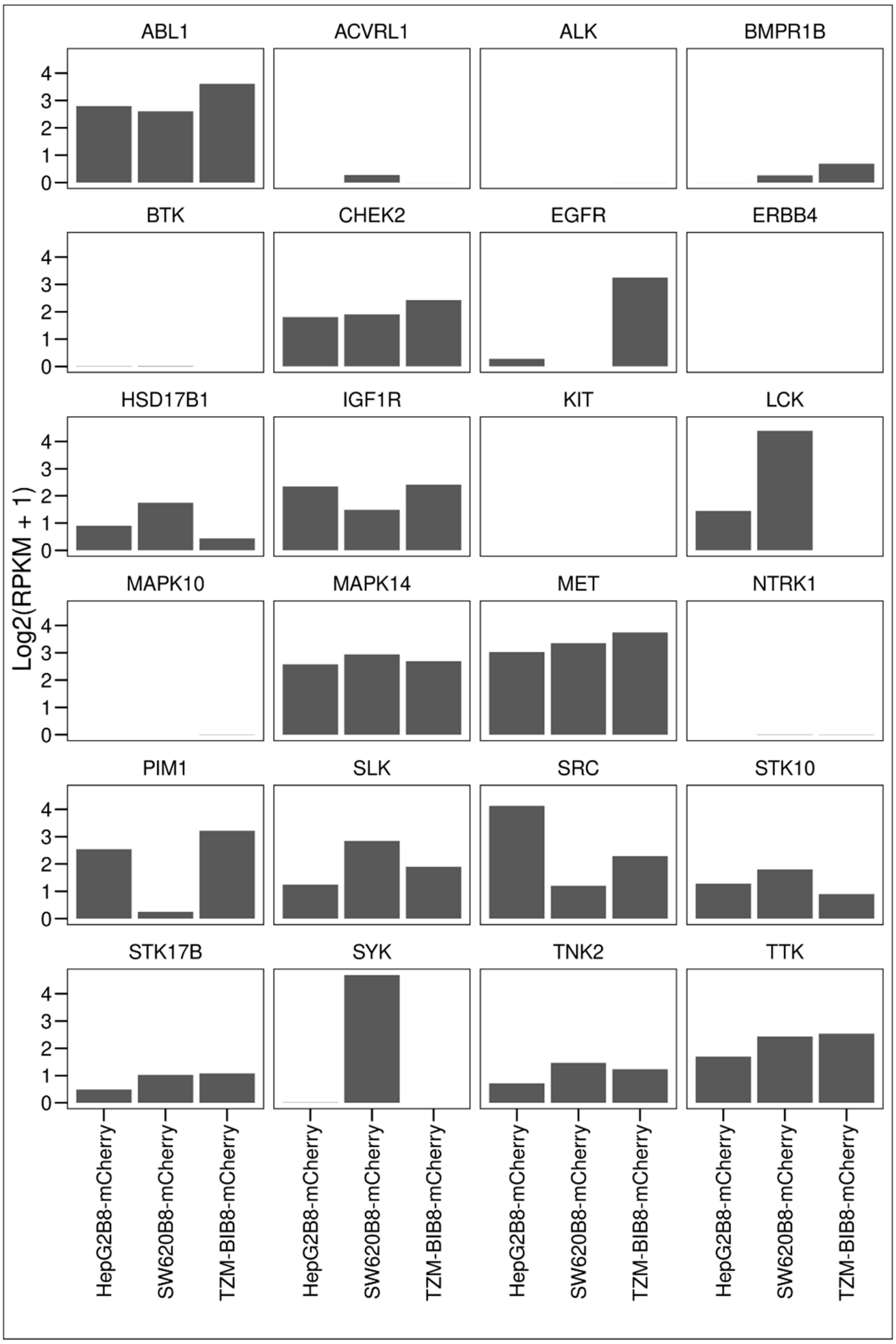

Supplementary Figure S11. The expression of EGFR gene and 23 Gefitinib off target genes in the modified cells.
