# Supplementary material for: A Cell-Based Platform for the Investigation of Immunoproteasome Subunit β5i Expression and Biology of β5i-Containing Proteasomes
Source: Cells. 2021 Nov 5;10(11):3049. doi: 10.3390/cells10113049 (PMC8616536; doi:10.3390/cells10113049)
Supplement: Supplementary file 1 [file cells-10-03049-s001.zip › Supplementary materials - Table S1.pdf]

Table S1. Primers used in the study.

Primer / Oligonucleotide	Sequence 5′ – 3′	Reference, Position (bp)
**gRNA Cloning into pDG461**		
gRNA_1_olig_top	CACCTGGCTTCCCGGTACTGGTGCAGC	NC_000006.12: 3691 - 3713
gRNA_1_olig_bottom	AAACGCTGCACCAGTACCGGGAAGCCA	NC_000006.12: 3691 - 3713
gRNA_2_olig_top	ACCGTCAATAATGGTGGTGGTGGCAGCGT	NC_000006.12: 3971 - 3995
gRNA_2_olig_bottom	TAAAACGCTGCCACCACCACCATTATTGA	NC_000006.12: 3971 - 3995
**Donor fragment cloning into pcDNA3.1(-) and pAL-2T**		
Left_HA_fwd	GGGAGACCCAAGCTGGCAGAGTCAGAA GAAGGGCAT	NC_000006.12: 3488 - 3507
Left_HA_rev	TGGCTTCCCGGTACTGGTGCAGCAGATCAC TGACATCTG	NC_000006.12: 3675 - 3713
Adapter_1_top	GTAGAAAGTACAGATGTCAGTGATCTGCT GCACCAGTACCGGGAAGCCAATCAAGGAT CCGGAGGTGGAGGTTCAGGA	-
Adapter_1_bottom	TCCTGAACCTCCACCTCCGGATCCTTGATTG GCTTCCCGGTACTGGTGCAGCAGATCACTGACAT CTGTACTTTCTAC	-
Insert_fwd	AGTACCGGGAAGCCAATCAAGGATCC GGAGGTGGAGGTTCAGG	-
Insert_rev_mCherry	CTGCCAAGCTGCCACCACCACCATTA CTTGTACAGCTCGTCCATGC	-
Adapter_2_top_mCherry	GCATGGACGAGCTGTACAAGTAATGGTG GTGGTGGCAGCTTGGCAGGTCTCC TCTGGGAGGTCTTG	-
Adapter_2_bottom_mCherry	CAAGACCTCCCAGAGGAGACCTGCCAAGC TGCCACCACCACCATTACTTGTACA GCTCGTCCATGC	-
Right_HA_fwd	TGGTGGTGGTGGCAGCTTGGCAG GTCTCCTCTGGGAGGT	NC_000006.12: 3978 - 4010
Right_HA_rev	CGAGTCTAGAGGGCCCGTTTAAACGCCGTT TTTCTTTATTCTACTTAGTGGG	NC_000006.12: 4184 - 4210
**Control and Sequencing**		
Genome control_fwd (Primer A)	CCATGTTACCAACATCTTCCTCTCC	NC_000006.12: 3146 - 3171
Control_mCherry_rev (Primer B)	CACCCTTGGTCACCTTCAGCTTGGC	-
Cherry_control_fwd (Primer C)	GCCACTACGACGCTGAGGTCAAGACC	-
Rev_control (Primer D)	GCCCCACCCAACAACCACAGGC	NG_028165.1: 9269-9248
Transcript_seq_fwd (Primer E)	CCTGTTTCCAAGACTCTGCC	NM_004159.5: 211-230

Transcript_seq_rev (Primer F)	TCCTCTGGCTGCTGAGCC	NM_004159.5: 1220-1203
qPCR_transgene_fwd (Primer G)	GAAGGAAGATGGTTGGGTGAAAG	NM_004159.5: 1007-1029
qPCR_transgene_rev (Primer H)	ACCTTGAAGCGCATGAACTCC	-
Exon6_control_fwd	GGTGAACAAGGTGATTGAG	NM_148919.3: 349-367
Control_RNA_mCherry_rev	GTACAGCTCGTCCATGCCG	-
SG_primer	TTTGTCTGCAGAATTGGC	-
SK_primer	TCTAGAACTAGTGGATC	-
M13_fwd	GTAAAACGACGGCCAGT	-
M13_rev	CAGGAAACAGCTATGAC	-
